# Improvement of osteogenic differentiation potential of placenta-derived mesenchymal stem cells by metformin via AMPK pathway activation

**DOI:** 10.1186/s13287-024-04014-6

**Published:** 2024-11-13

**Authors:** Sirikul Manochantr, Ladda Meesuk, Nuengruethai Chadee, Jintamai Suwanprateeb, Chairat Tantrawatpan, Pakpoom Kheolamai

**Affiliations:** 1 Division of Cell Biology, Department of Preclinical Sciences, Faculty of Medicine, Thammasat University, KhlongLuang, Pathum Thani 12120 Thailand; 2 Center of Excellence in Stem Research and Innovation, Thammasat University, KhlongLuang, Pathum Thani 12120 Thailand; 3 Biomedical Engineering Research Unit, National Metal and Materials Technology Center (MTEC), Ministry of Science and Technology, Khlong Nueng, 12120 Pathum Thani Thailand

**Keywords:** Metformin, Mesenchymal stem cells, Osteogenic differentiation, Diabetes mellitus

## Abstract

**Background:**

Placenta-derived human mesenchymal stem cells (PL-MSCs) have gained a lot of attention in the field of regenerative medicine due to their availability and bone-forming capacity. However, the osteogenic differentiation capacity of these cells remains inconsistent and could be improved to achieve greater efficiency. Although metformin, a widely used oral hypoglycemic agent, has been shown to increase bone formation in various cell types, its effect on osteogenic differentiation of PL-MSCs has not yet been investigated. Therefore, the objective of this study was to examine the effect of metformin on the osteogenic differentiation capacity of PL-MSCs and the underlying mechanisms.

**Methods:**

The PL-MSCs were treated with 0.5 to 640 µM metformin and their osteogenic differentiation capacity was examined by an alkaline phosphatase (ALP) activity assay, Alizarin red S staining and expression levels of osteogenic genes. The role of adenosine 5′-monophosphate-activated protein kinase (AMPK) signaling in mediating the effect of metformin on the osteogenic differentiation capacity of PL-MSCs was also investigated by determining levels of phosphorylated AMPK (pAMPK)/AMPK ratio and by using compound C, an AMPK inhibitor.

**Results:**

The results showed that 10–160 µM metformin significantly increased the viability of PL-MSCs in a dose- and time-dependent manner. Furthermore, 80–320 µM metformin also increased ALP activity, matrix mineralization, and expression levels of osteogenic genes, runt-related transcription factor 2 (*RUNX2*), osterix (*OSX*), osteocalcin (*OCN*) and collagen I (*COL1*), in PL-MSCs. Metformin increases osteogenic differentiation of PL-MSCs, at least in part, through the AMPK signaling pathway, since the administration of compound C inhibited its enhancing effects on ALP activity, matrix mineralization, and osteogenic gene expression of PL-MSCs.

**Conclusions:**

This study demonstrated that metformin at concentrations of 80–320 μM significantly enhanced osteogenic differentiation of PL-MSCs in a dose- and time-dependent manner, primarily through activation of the AMPK signaling pathway. This finding suggests that metformin could be used with other conventional drugs to induce bone regeneration in various bone diseases. Additionally, this study provides valuable insights for future osteoporosis treatment by highlighting the potential of modulating the AMPK pathway to improve bone regeneration.

**Supplementary Information:**

The online version contains supplementary material available at 10.1186/s13287-024-04014-6.

## Introduction

Mesenchymal stem cells (MSCs) have the ability to undergo extensive self-renewal and differentiate into several mesodermal lineages, such as osteoblasts, chondrocytes, and adipocytes [1]. Due to their regenerative capacity and low immunogenicity, MSCs have attracted significant interest in regenerative medicine. MSCs can be isolated from numerous tissues, such as bone marrow, adipose tissue, placenta, amnion, and umbilical cord [2, 3]. Among these tissues, bone marrow has been a typical source of MSCs for clinical application. However, bone marrow aspiration is invasive and the amount of bone marrow-derived MSCs (BM-MSCs) decreases with age [4]. As a result, there has been a growing focus on finding alternative sources of MSCs that are readily available and can be collected non-invasively, such as the placenta [5].

Placenta-derived human MSCs (PL-MSCs) can be obtained non-invasively and can be successfully recovered after extended periods of cryopreservation. PL-MSCs have greater proliferation capacity than BM-MSCs [2] and exhibit several key functions, particularly in the context of osteogenic differentiation, which makes them valuable for regenerative medicine and bone tissue engineering [6, 7]. PL-MSCs play a vital role in bone formation by differentiating into osteoblasts, producing bone matrix, and increasing matrix mineralization. Furthermore, PL-MSCs also secrete many bioactive molecules that increase osteogenesis by recruiting and stimulating endogenous stem cells, promoting angiogenesis [8, 9], and creating an anti-inflammatory environment that is vital for bone regeneration [10, 11]. The properties make PL-MSCs a promising candidate for clinical applications in the treatment of various bone diseases, fractures, and osteoporosis, which has been highlighted in previous studies [12, 13]. However, the osteogenic differentiation capacity of PL-MSCs remains inconsistent and could be improved to achieve greater efficiency.

Metformin, a widely used oral hypoglycemic agent in patients with Type 2 diabetes mellitus (T2DM), reduces blood glucose by inhibiting hepatic gluconeogenesis and improves glucose uptake by activating adenosine monophosphate-activated protein kinase (AMPK) in liver and peripheral tissue of diabetic patients [14, 15]. In addition to the glucose-lowering effect, metformin has been shown to have beneficial effects on bone metabolism and bone formation in diabetic patients [16]. As an activator of AMPK, metformin promotes bone formation by increasing osteoblast differentiation, while inhibiting bone resorption by inhibiting osteoclast function [17]. Metformin increased the formation of trabecular bone in primary osteoblasts derived from rat calvaria [18] and rat osteosarcoma cells [19]. It also induces osteogenic differentiation and matrix mineralization in bone marrow progenitor cells of streptozotocin-induced diabetic rats by activating AMPK and RUNX2 [20]. Metformin also inhibits the generation of advanced glycation end products (AGEs), leads to increased bone turnover and improved bone health in adult women without T2DM. Already recognized as a safe and effective treatment for diabetes, metformin has potential for long-term prevention and treatment of osteoporosis [21]. Although the aforementioned research indicates that metformin facilitates osteoblastic differentiation in various cell types by activating the AMPK signaling pathway, its effect on osteogenic differentiation of PL-MSCs has not yet been investigated. Therefore, the objective of this study was to examine the effect of metformin on the osteogenic differentiation capacity of PL-MSCs and the role of the AMPK signaling pathway in the osteogenic effects induced by metformin. The acquired results could be used to improve the bone-forming capacity of PL-MSCs for stem cell-based tissue engineering and provide clues for future osteoporosis treatment by modulating the AMPK pathway.

## Materials and methods

### Isolation and culture of PL-MSCs

The study was approved by the Human Ethics Committee of Thammasat University (Medicine) [Approval number: 037/2021]. Placental tissues were obtained from pregnant women after normal delivery with written informed consent. The MSCs were isolated from the placenta and characterized as previously described [11]. The tissues were intensively washed with washing buffer [1X phosphate buffered saline (PBS) containing 100 units/ml of penicillin and 100 µg/ml streptomycin (GibcoBRL, USA)] and minced into small pieces. Subsequently, the tissues were digested with 0.5% trypsin–EDTA (Invitrogen, USA) at 37 °C for 2 h with shaking. After washing twice with washing buffer, the cells were cultured with complete DMEM medium [Dulbecco's Modified Eagle’s Medium (DMEM; GibcoBRL, USA) containing 10% fetal bovine serum (FBS; HyClone™, USA), 1% GlutaMAX™ (GibcoBRL, USA), 100 units/ml of penicillin and 100 µg/ml streptomycin (GibcoBRL, USA)] in 25-cm^2^ culture flasks (Corning costar®, China). The cultures were maintained at 37 °C in a humidified tissue culture incubator with 5% CO_2_. The culture medium was changed every 3 days. Plastic-adherent cells with approximately 80–90% confluence were subcultured with 0.25% trypsin–EDTA (GibcoBRL, USA), cryopreserved in a freezing medium, and stored in liquid nitrogen for further experiments.

### Characterization of PL-MSCs

#### Morphology

The morphology of PL-MSCs kept in an incubator was observed by inverted microscopy (Nikon Eclipse Ts2R-FL, Japan) every day. Adherent cells were sub-passaged when they reached 80–90% confluence.

#### Immunophenotype

The PL-MSCs in passages 3 to 5 were trypsinized with 0.25% trypsin–EDTA and washed with 1X PBS. After centrifugation, 5 × 10^5^ cells were resuspended in 50 µl of 1X PBS and incubated with 5 µl of fluorescein isothiocyanate (FITC) or phycoerythrin (PE)-conjugated antibody against HLA-DR (Bio Legend, USA), cluster of differentiation (CD) 34 (BioLegend, USA), CD45 (BioLegend, USA), CD73 (BioLegend, USA), CD90 (BioLegend, USA) and CD105 (BD Bioscience, USA) at 4 °C for 30 min in the dark. After washing with 1X PBS, the cells were fixed with 1% paraformaldehyde in PBS. The positive cells were identified by comparing with isotype-matched controls [FITC-conjugated mouse immunoglobulin G1 (IgG1) and PE-conjugated mouse immunoglobulin G2a (IgG2a)]. At least twenty thousand labeled cells were acquired and analyzed using flow cytometry (DxFLEX flow cytometer, Beckman Coulter, USA) and CytExpert software (DxFLEX flow cytometer, Beckman Coulter, USA).

#### Osteogenic differentiation

After trypsinization, PL-MSCs in passages 3–5 were seeded in 6-well plates (Corning costar®, China) at a density of 4.5 × 10^3^ cells/cm^2^. Cells were cultured with complete DMEM medium at 37° C in a humidified incubator with 5% CO_2_ until they reached 80% confluence. After washing with 1X PBS, 1.5 ml of osteogenic differentiation medium [complete DMEM medium supplemented with 100 nM dexamethasone (Sigma-Aldrich, USA), 10 mM β-glycerophosphate (Sigma-Aldrich, USA), and 50 µg/ml ascorbic acid (Sigma-Aldrich, USA)] was added. The medium was changed every 3 days. On day 28, the cultured cells were fixed with 10% formaldehyde and stained with 40 mM Alizarin Red S (Sigma-Aldrich, USA) for 20 min at room temperature. The cells were washed with distilled water and observed under an inverted microscope (Nikon Eclipse Ts2R-FL, Japan). Differentiated MSCs were visualized as red cells correlated with calcium deposits in the cells. For the control, cells were cultured in a complete DMEM medium without any osteogenic stimuli, performed in parallel with the experimental group, and stained in the same way.

#### Adipogenic differentiation

After trypsinization, PL-MSCs in passages 3–5 were cultured with complete DMEM medium in 6-well plates (Corning costar®, China) at a density of 4.5 × 10^3^ cells/cm^2^. Cells were kept at 37 °C in a humidified incubator with 5% CO_2_ until they reached 80% confluence. Subsequently, cells were washed with 1X PBS and 1.5 ml of adipogenic differentiation medium [complete DMEM medium supplemented with 2.5 mM glucose (Sigma-Aldrich, USA), 0.5 mM isobutyl methylxanthine (IBMX; Sigma-Aldrich, USA), 1 µM dexamethasone (Sigma-Aldrich, USA), 10 µM insulin (Sigma-Aldrich, USA) and 0.2 mM indomethacin (Sigma-Aldrich, USA)] was added. The medium was changed every 3 days. On day 28, cells were fixed with 37% formalin solution vapor and stained with 0.5% oil red O (Sigma-Aldrich, USA) in 6% isopropanol. The cells were then washed with distilled water and observed by inverted microscopy (Nikon Eclipse Ts2R-FL, Japan). Lipid droplets in differentiated PL-MSCs were marked red, in correlation with lipid accumulation in the cells. For the control group, cells were maintained in the complete medium without adipogenic stimuli, performed in parallel with the experimental group, and stained in the same manner.

#### Chondrogenic differentiation

After trypsinization, PL-MSCs in passages 3–5 were initially cultured with complete DMEM medium at a density of 3 × 10^6^ cells/cm^2^ in 96-well U-bottom cell culture plates (Jet Biofil, China) at 37°C with 5% CO_2_ overnight. The medium was removed and MSCgo^TM^ Chondrogenic XF medium (Sartorius, Germany) was added. On day 21, the spherical mass was fixed with 10% formalin solution for 30 min at room temperature. The mass was then stained with a 1% Alcian Blue Solution (HiMedia, India) in a dark environment at room temperature overnight. The stained sample was examined using inverted microscopy (Nikon Eclipse Ts2R, Japan). For the control, PL-MSCs were cultured in a complete DMEM medium without chondrogenic stimuli, and processed similarly to cells in differentiation medium.

### Cytotoxicity assay

To evaluate the effect of metformin on viability of PL-MSCs, 1 × 10^3^ PL-MSCs were cultured with complete DMEM medium in a 96-well plate (SPL Life Science, Korea) at 37 °C in a 5% CO_2_ humidified incubator. Cells were allowed to adhere to the plate for 24 h. Subsequently, the cells were washed with 1X PBS and treated with 100 µl of complete DMEM medium containing 0.5, 10, 40, 80, 160, 320, and 640 µM metformin. The control was cultured in complete DMEM medium without metformin. The cultures were maintained for 14 days and cell viability was measured every 2 days using an MTT assay (Sigma-Aldrich, USA). Briefly, 2 mg/ml of MTT solution (3- (4,5-Dimethylthiazol-2-yl) -2,5-Diphenyltetrazolium bromide) in complete DMEM medium was added to each well and incubated at 37°C in a humidified incubator with 5% CO_2_ for 4 h. Subsequently, the entire solution was removed and the purple formazan crystals were dissolved in 100 µl of dimethyl sulfoxide (DMSO, VWR BDH Chemicals, France). The absorbance was measured at 570 nm using a microplate reader (BioTex, USA). The percentage of cell viability was calculated using the following equation:

$${\text{Cell}}\;{\text{viability}}\left( {\% \;{\text{of}}\;{\text{the}}\;{\text{control}}} \right) = \frac{{{\text{OD}}\left( {{\text{sample}}} \right) - {\text{OD}}\left( {{\text{blank}}} \right)}}{{{\text{OD}}\left( {{\text{control}}} \right) - {\text{OD}}\left( {{\text{blank}}} \right)}} \times 100$$

### Osteogenic differentiation assay

The osteogenic differentiation potential of PL-MSCs was evaluated using an osteogenic differentiation assay as previously described [22]. The PL-MSCs were seeded in 6-well plates (Corning costar®, China) at a density of 4.5 × 10^3^ cells/cm^2^ and cultured with complete DMEM medium at 37 °C in a humidified incubator with 5% CO_2_ until 80% confluence. The cells were then washed with 1X PBS and 1.5 ml of osteogenic differentiation medium containing 10, 40, 80, 160, and 320 µM metformin was added. PL-MSCs cultured with osteogenic differentiation medium without metformin were used as a control. On days 7, 14, 21, and 28, the expression of osteogenic genes, the alkaline phosphatase (ALP) activity assay, and the Alizarin Red S staining were performed as follows:

#### Expression of osteogenic genes

Total RNA extraction was performed using TRIzol® reagent (Invitrogen, USA). Total RNA was reverse transcribed into cDNA using iScript™ reverse transcription Supermix for RT-qPCR (Bio-Rad, USA) according to the manufacturer's instructions. Quantitative real-time reverse transcription-polymerase chain reaction (qRT-PCR) was used to measure the expression of osteogenic markers (RUNX2, Osterix, Osteocalcin and Collagen I) in metformin-treated PL-MSCs compared to untreated PL-MSCs. Gene expression was quantified using the SYBR Green PCR Master Mix (Applied Biosystems, USA). The cycling program was set as follows: thermal activation at 95 °C for 30 s and 40 cycles of PCR (melting at 95 °C for 15 s, annealing at 60 °C for 60 s). The primer sequences are shown in Table 1. Relative expression was evaluated using the 2^ΔΔCt^ method and normalized by the Ct of glyceraldehyde-3-phosphate dehydrogenase (GAPDH). All reactions were performed in at least triplicate and analysis was performed using an Applied Biosystems StepOne™ Plus real-time PCR system and StepOne™ Software version 2.3 (Applied Biosystems; ABI, USA).

**Table 1:** Sequence of primers used for qRT-PCR

Gene	Forward primer	Reverse primer
RUNX2	5′-GACAGCCCCAACTTCCTGTG-3’	5′-CCGGAGCTCAGCAGAATAAT-3’
Osterix	5′-TGCTTGAGGAGGAAGTTCAC-3’	5′-CTGCTTTGCCCAGAGTTGTT-3’
Osteocalcin	5′-CTCACACTCCTCGCCCTATT-3’	5′-TCAGCCAACTCGTCACAGTC-3’
Collagen I	5′-CCTGGATGCCATCAAAGTCT-3’	5′-AATCCATCGGTCATGCTCTC-3’
AMPK	5′-TGAGAGCTGGAACGGACTTT-3’	5′-CTGGTAGCTTGGCTCAGTCC-3’
GAPDH	5′-CAATGACCCCTTCATTGACC-3’	5′-TTGATTTTGGAGGGATCTCG-3’

#### Alkaline phosphatase activity assay

Alkaline phosphatase (ALP) activity was measured using the SensoLyte® pNPP Alkaline Phosphatase Assay Kit (Ana Spec, USA). On days 7, 14, 21, and 28 after treatment, the culture medium was removed, the cell monolayer was washed with 1X PBS and cell lysis was performed using 200 µl of lysis buffer (0.1 M glycine, 1% Nonidet P-40, 1 mM MgCl_2_ and 1 mM ZnCl_2_, pH 9.6). The cells were then scratched off the plastic surface and incubated on ice for 20 min. After centrifugation at 2500 × g, 4 °C for 10 min, the supernatant was collected and transferred to a new 96-well plate (SPL Life Science, Korea). Then, 50 µl of p-nitrophenyl phosphate (pNPP) was added to each well, mixed by gently shaking the plate for 30 s, and incubated for 30 min at 37°C. The final solution, a yellow-colored product, was measured using a microplate reader (BioTex, USA) at an absorbance of 405 nm. The ALP activity in each sample was calculated by comparing the measured OD values with a standard curve generated from 0 to 200 ng/ml of the standard ALP solution. Each test condition was performed in triplicate and normalized with the concentrations of total cellular proteins measured by the Bradford assay (Bio-Rad, USA).

#### Alizarin Red staining

The formation of calcium-containing mineral nodules during osteogenic differentiation of metformin-treated PL-MSCs compared to untreated PL-MSCs was determined using Alizarin Red S staining. After washing with 1X PBS, the PL-MSCs were fixed with 10% formaldehyde at room temperature for 15 min. The cells were then washed with distilled water and stained with 40 mM Alizarin Red S (Sigma-Aldrich, USA) for 20 min at room temperature. The cells were washed with distilled water and observed under an inverted microscope (Nikon Eclipse Ts2R-FL, Japan).

For the quantification of Alizarin Red S staining, 400 µl of 10% acetic acid was added to each well and the plate was incubated at room temperature for 30 min. The monolayer was then scratched off the plastic surface, transferred to the microcentrifuge tube, and sealed with parafilm to prevent evaporation. After vortexing for 30 s, the samples were heated at exactly 85 °C for 10 min, and incubated on ice for 5 min. The lysate was centrifuged at 20,000 x g for 15 min and 300 µl of the supernatant was transferred to a new 1.5 ml microcentrifuge tube. Subsequently, 150 µl of 10% ammonium hydroxide was added to neutralize the acid. The samples, a yellow-colored product, were measured using a microplate reader (BioTex, USA) at an absorbance of 405 nm.

### AMPK activity assay

To study the relationship between AMPK activation and osteogenic differentiation potential of metformin-treated PL-MSCs, we investigated the levels of AMPK and phosphorylated AMPK in metformin-treated PL-MSCs and compared them to levels in untreated PL-MSCs. PL-MSCs were seeded in 6-well plates (Corning costar®, China) at a density of 4.5 × 10^3^ cells/cm^2^ and cultured with complete DMEM medium at 37 °C in a humidified incubator with 5% CO_2_ until 80% confluence. The cells were then washed with 1X PBS and 1.5 ml of osteogenic differentiation medium containing 10, 40, 80, 160, and 320 µM metformin was added. To determine the role of the AMPK signaling pathway in metformin-induced osteogenic differentiation, 10 µM Compound C (6-[4-(2-Piperidin-1-ylethoxy) phenyl]-3-pyridin-4-ylpyrazolo [1,5-a] pyrimidine; the AMPK inhibitor) was added to the culture. PL-MSCs cultured in osteogenic differentiation medium without metformin were used as a control. Western blot and qRT-PCR were performed to examine the expression of AMPK after metformin treatment.

#### Western blot analysis

The protein was extracted using RIPA reagent (Bio-Rad, USA) containing a protease and phosphatase inhibitor cocktail (Cell Signaling Technology, USA). The protein concentration was determined using a Bradford assay (Bio-Rad, USA). The total protein (20 µg) from each sample was diluted with the 3X reducing SDS loading buffer (Cell Signaling Technology, USA), and a molecular weight marker (Abcam, USA) was then separated by electrophoresis using a 12% sodium dodecyl sulfate–polyacrylamide gel electrophoresis (SDS-PAGE). The electrophoresis was carried out at 120 V for 90 min. The proteins on SDS-PAGE gels were transferred to a nitrocellulose membrane (0.45 µm pore-size; Bio-Rad, USA) using a mini trans-blot electrophoretic transfer cell machine (Bio-Rad, USA) at 120 V for 90 min. Following transfer, the membranes were gently stained using 0.1% (w/v) Ponceau S in 5% acetic acid. Next, the membranes were carefully cut to facilitate the incubation process with different primary antibodies. The membranes were then blocked with 5% skim milk in TBS at room temperature for 1 h. Next, the membranes were incubated with primary antibodies, including rabbit anti-human AMPKα (1:1000 dilution; Cell Signaling Technology, USA), rabbit anti-human phospho-AMPKα (Thr172) (1:1000 dilution; Cell Signaling Technology, USA) and rabbit anti-human actin (1:10,000 dilution; Proteintech, USA) at 4 °C, overnight. The membranes were washed with 0.1% Tween-20 in TBS 3 times. Subsequently, the membranes were incubated with peroxidase-conjugated IgG fraction monoclonal mouse anti-rabbit secondary antibody (1:10,000 dilution; Jackson ImmunoResearch, USA) for 1 h at room temperature. The proteins were visualized using enhanced chemiluminescence (ECL) using Clarity™ Western ECL Substrate (Bio-Rad, USA). The signals were captured with Amersham Imager 600 (GE Healthcare Life Sciences). For quantitation, the membrane was scanned, and the digital image was saved in black-and-white JPEG format. The digital image was analyzed using ImageJ software available in the public domain at https://imagej.nih.gov/ij/ download.html. Briefly, the digital image was opened in ImageJ software, and the bands were outlined using a squared region tool and added to the ROI (Region of Interest) manager. The image was inverted, and the analysis tool was used to measure the integral optical density (IOD) of the individual bands. The intensity of the protein band was expressed as a ratio to β-actin band intensity.

#### Quantitative real-time RT-PCR

For qRT-PCR, cells were washed with 1X PBS and total RNA extraction was performed using TRIzol® reagent (Invitrogen, USA). Total RNA was reverse transcribed into cDNA using iScript™ reverse transcription Supermix for RT-qPCR (Bio-Rad, USA) according to the manufacturer's instructions. Gene expression was quantified using the SYBR Green PCR Master Mix (Applied Biosystems, USA). The cycling program was set as follows: thermal activation at 95 °C for 30 s and 40 cycles of PCR (melting at 95 °C for 15 s, annealing at 60 °C for 60 s). The primer sequences are shown in Table 1. Relative expression was evaluated using the 2^ΔΔCt^ method and normalized by the Ct of glyceraldehyde-3-phosphate dehydrogenase (GAPDH). The qRT-PCR analyzes were performed using an Applied Biosystems StepOne™ Plus real-time PCR system and StepOne™ software version 2.3 (Applied Biosystems; ABI, USA).

#### Inhibition of AMPK activity using compound C

PL-MSCs were seeded in 6-well plates (Corning costar®, China) at a density of 4.5 × 10^3^ cells/cm^2^ and cultured with complete DMEM medium at 37 °C in a humidified incubator with 5% CO_2_ until 80% confluence. The cells were then washed with 1X PBS and cultured with osteogenic differentiation medium containing 10, 40, 80, 160, and 320 µM metformin with or without 10 µM of Compound C (Sigma-Aldrich, USA). The medium was changed every 3 days. The osteogenic differentiation potential of PL-MSCs after compound C treatment was determined by a gene expression study, an ALP activity assay, and Alizarin Red S staining, as mentioned above.

### Statistical analysis

Data were analyzed and presented as mean ± standard error of the mean (SEM). Statistical significance was assessed using one-way analysis of variance (ANOVA) using SPSS version 26, developed by SPSS, Inc. A significance level of *p* < 0.05 was considered statistically significant.

## Results

### The characteristics of PL-MSCs

**Fig. 1:**
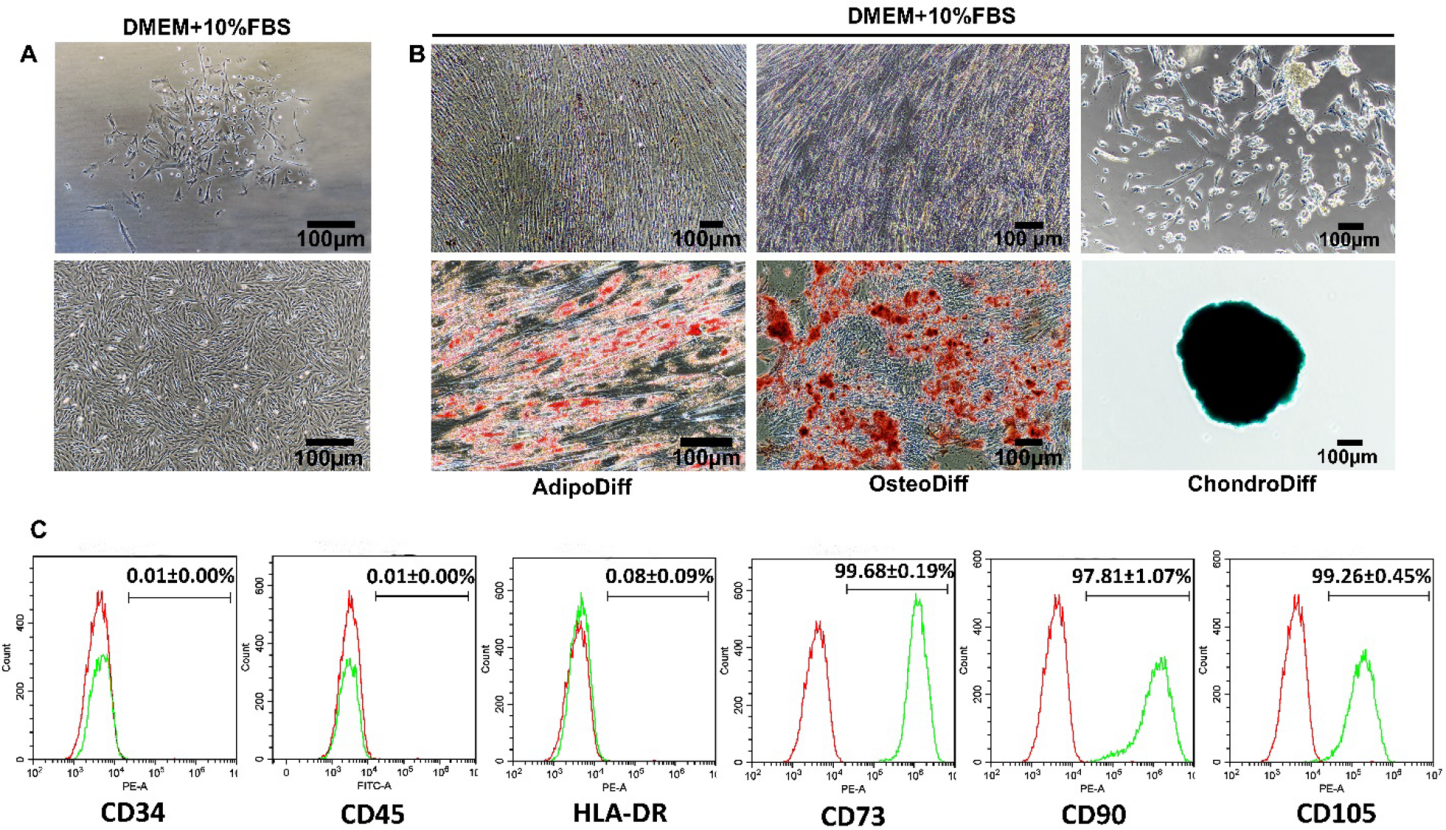
The characteristics of MSCs derived from human placenta (PL-MSCs) **A** The spindle morphology of the PL-MSCs cultured in DMEM + 10% FBS on day 7 (upper) and in passages 3 (lower). **B** The positive staining of oil red O, Alizarin Red S and Alcian Blue confirmed the adipogenic differentiation, osteogenic differentiation and chondrogenic differentiation potentials of PL-MSCs, respectively, compared to the control cultured in DMEM + 10% FBS. **C** Flow cytometry analysis showed positive expression of MSC markers, CD73, CD90, CD105, and negative expression of hematopoietic markers, CD34, CD45, and HLA-DR in PL-MSCs. Data are presented as mean ± SEM (n = 3). Scale bar = 100 μm

PL-MSCs exhibited spindle-shaped morphology (Fig. 1A), expressed typical MSC surface markers, CD73 (99.68 ± 0.19%), CD90 (97.81 ± 1.07%), and CD105 (99.26 ± 0.45%) but did not express hematopoietic surface markers, CD34 (0.01 ± 0.00%), CD45 (0.01 ± 0.00%), and HLA-DR (0.08 ± 0.09) (Fig. 1C). Under appropriate culture conditions, these PL-MSCs can also differentiate into adipocytes, osteoblasts, and chondrocytes (Fig. 1B), as demonstrated by oil red O, Alizarin Red S and Alcian Blue staining, respectively. The PL-MSCs can be expanded for at least 15 passages in culture. These results show that the characteristics of the PL-MSCs established in this study match the criteria of the International Society for Cell & Gene Therapy (ISCT) [23].

### Effect of metformin on the viability of PL-MSCs

**Fig. 2:**
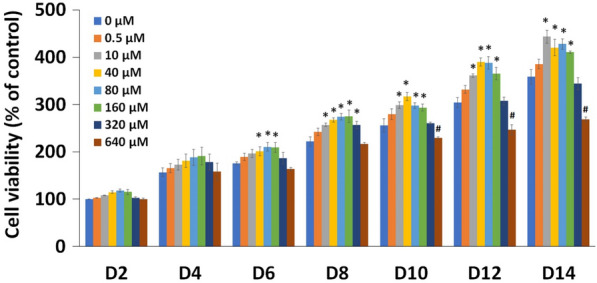
The MTT assay demonstrated the effect of metformin on the viability of PL-MSCs. Data are presented as mean ± SEM (n = 3). Statistical significance was tested using the pair T-test. *Significant increase at *p*-value ≤ 0.05 compared to untreated PL-MSCs (0 µM). ^#^Significant decrease at *p*-value ≤ 0.05 compared to untreated PL-MSCs (0 µM)

The effect of metformin on the viability of PL-MSCs was determined by an MTT assay. The result showed that metformin, at concentrations of 10 to 160 µM, significantly increased the viability of PL-MSCs in a dose- and time-dependent manner (Fig. 2). Significant cytotoxicity of metformin was observed in PL-MSCs only when the concentration was greater than 320 µM (Fig. 2). Therefore, concentrations of metformin ranging from 10 to 320 µM were selected for subsequent experiments.

### Effect of metformin on osteogenic differentiation of PL-MSCs

The osteogenic differentiation of metformin-treated PL-MSCs was evaluated using an osteogenic gene expression assay, an alkaline phosphatase (ALP) activity assay, and Alizarin Red S staining. The results are as follows:

#### Effect on the expression of osteogenic genes

**Fig. 3:**
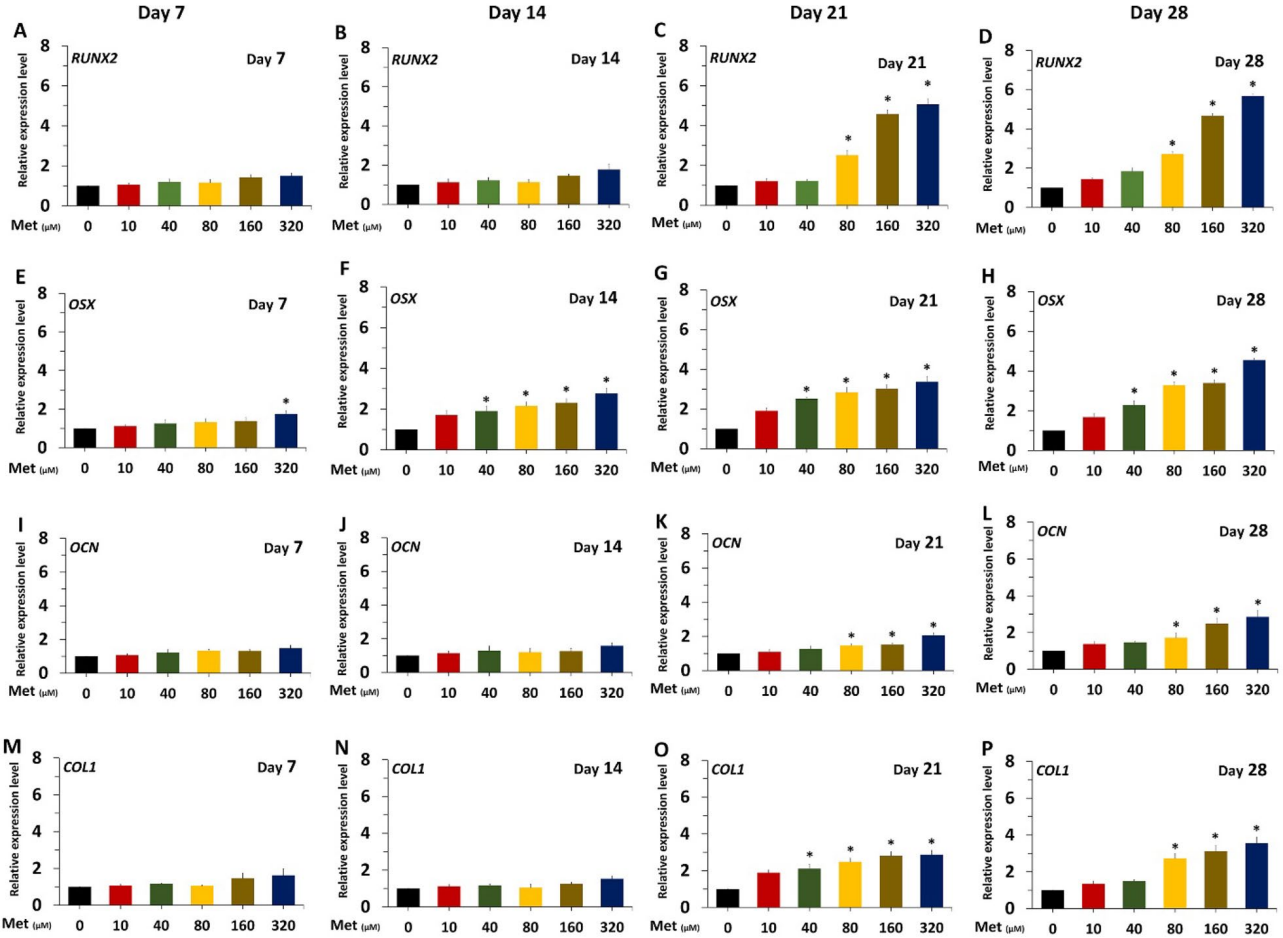
Quantitative real-time RT-PCR demonstrated the expression of osteogenic genes, *RUNX2*, osterix (*OSX*), osteocalcin (*OCN*) and collagen I (*COL1*) in PL-MSCs treated with various concentrations of metformin on days 7, 14, 21 and 28. Data are presented as mean ± SEM (n = 3). *p-value ≤ 0.05 compared to untreated PL-MSCs (0 µM)

The expression of osteogenic genes, including *RUNX2, *osterix (*OSX*), osteocalcin (*OCN*) and collagen I (*COL1*) in metformin-treated PL-MSCs was determined by qRT-PCR on days 7, 14, 21, and 28 after osteogenic induction culture. The results showed that metformin increased the expression levels of all osteogenic genes examined in a dose- and time-dependent manner (Fig. 3). Metformin at 320 µM, the highest concentration examined, increased the expression levels of *RUNX2, OSX, OCN* and *COL1* by approximately 5.7, 4.5, 2.8, and 3.6 fold, respectively, compared to the untreated control at the end of the culture on day 28 (Fig. 3).

#### Effect on alkaline phosphatase activity

**Fig. 4:**
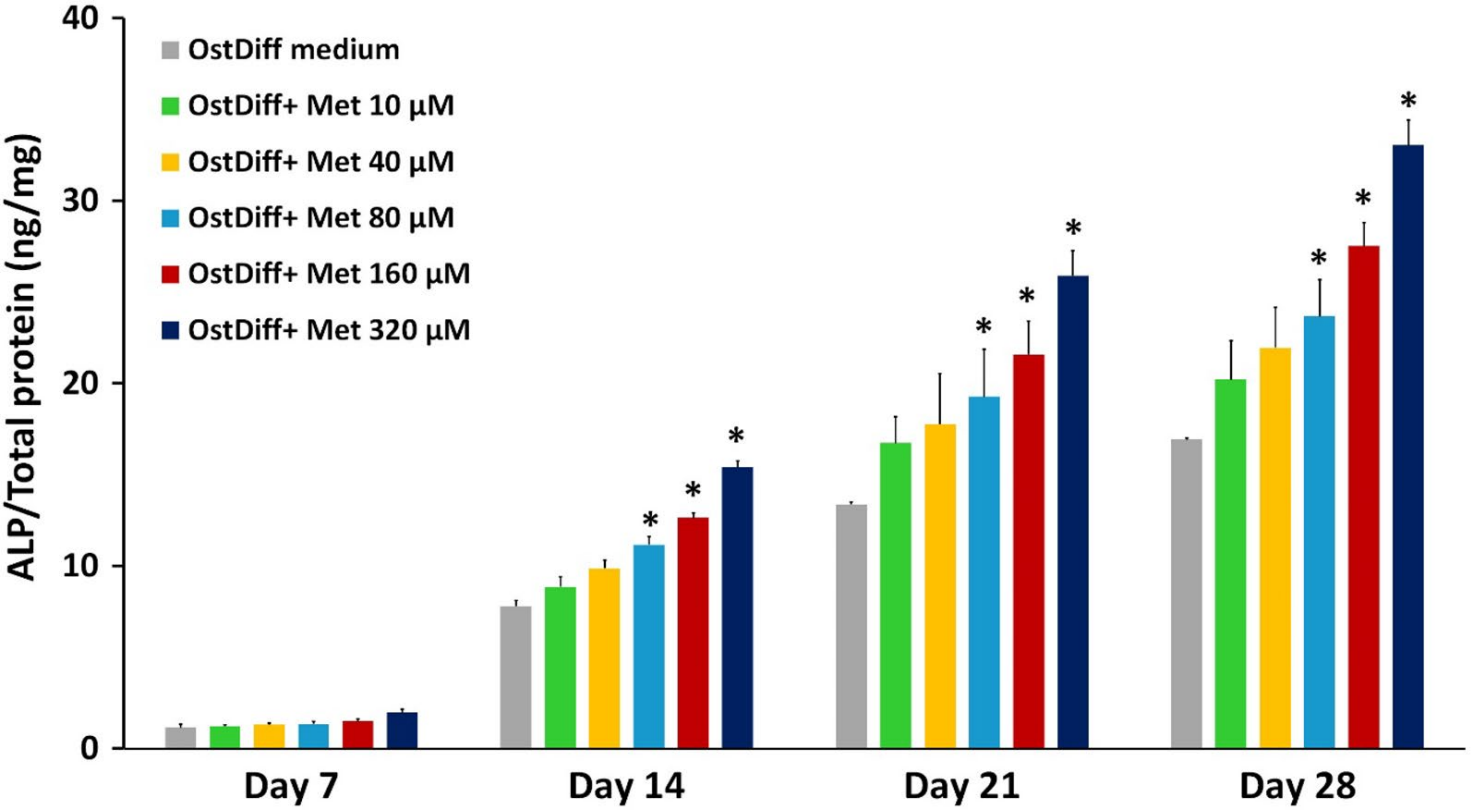
Alkaline phosphatase activity of metformin-treated PL-MSCs cultured in osteogenic differentiation medium for 28 days. PL-MSCs cultured in an osteogenic differentiation medium (OstDiff medium) without metformin serve as controls. Data are presented as mean ± SEM (n = 3). *p-value ≤ 0.05 compared to control

The ALP activity in metformin-treated PL-MSCs was determined by an ALP activity assay. Consistent with the gene expression study, metformin at concentrations of 80 to 320 µM significantly increased ALP activity in PL-MSCs compared to the untreated control in a dose- and time-dependent manner (Fig. 4). The positive effect of metformin on ALP activity was initially observed on day 14 of culture and continued to be observed until the end of culture (Fig. 4).

#### Effect on matrix mineralization

**Fig. 5:**
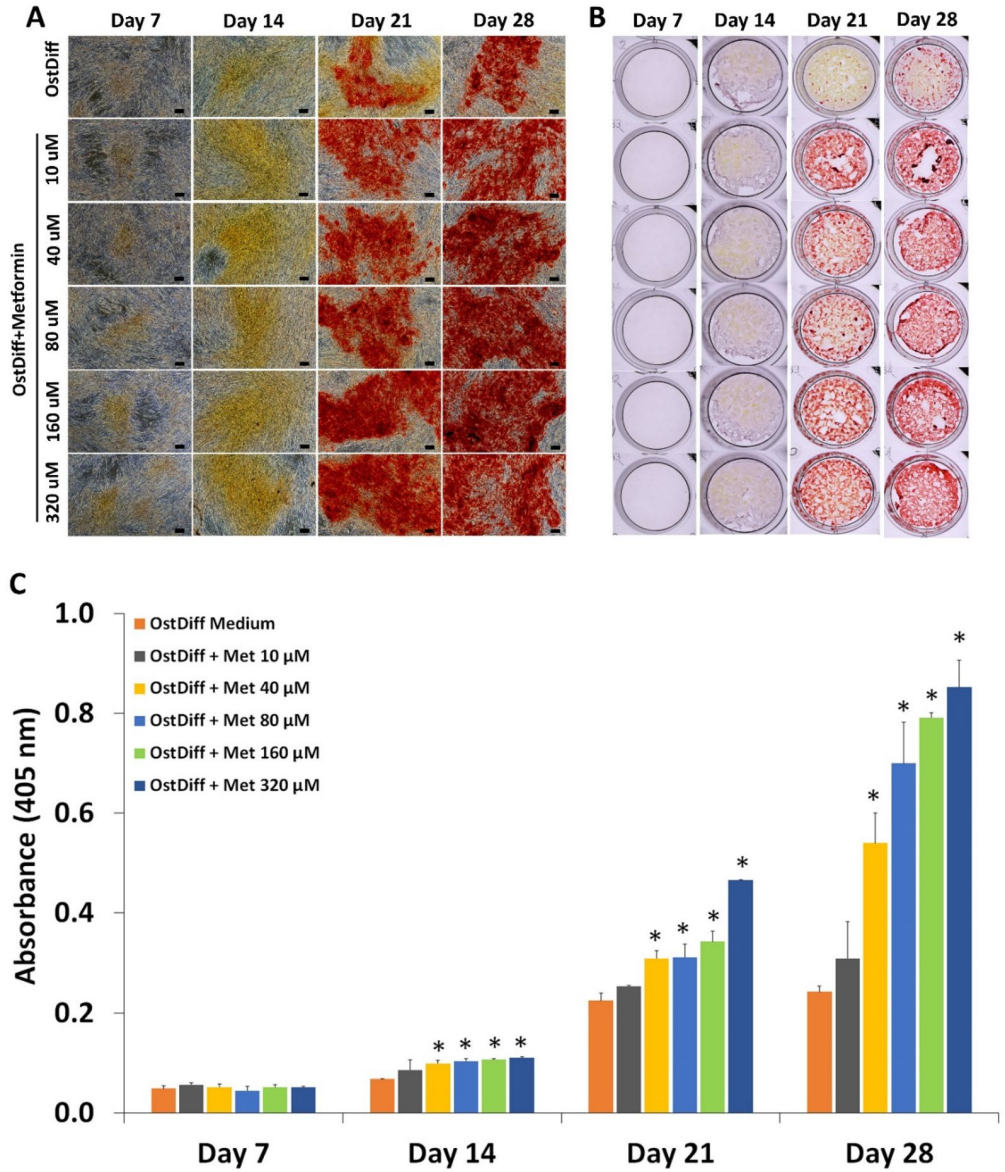
Alizarin Red S staining of metformin-treated PL-MSCs in osteogenic differentiation medium for 28 days. **A** Positive Alizarin Red S staining of metformin-treated PL-MSCs in osteogenic differentiation medium compared to PL-MSCs cultured in osteogenic differentiation medium (OstDiff) without metformin. Scale bar = 100 µm. **B** Whole well imaging of metformin-treated PL-MSCs in osteogenic differentiation medium compared PL-MSCs cultured in osteogenic differentiation medium (OstDiff) without metformin. **C** Quantification of Alizarin Red S staining. Data are presented as mean ± SEM (n = 3). *p-value ≤ 0.05 compared to PL-MSCs cultured in osteogenic differentiation medium (OstDiff medium) without metformin

Alizarin Red S staining was performed on days 7, 14, 21, and 28 to assess matrix mineralization in metformin-treated PL-MSCs. Matrix mineralization was initially observed in metformin-treated PL-MSCs cultured on day 21 of osteogenic induction and increased toward the end of culture, day 28 (Fig. 5A, B). Similarly to its effect on ALP activity, quantification of Alizarin Red S staining showed that metformin, at concentrations of 80 to 320 µM, significantly increased matrix mineralization in PL-MSCs compared to the untreated control in a dose- and time-dependent manner (Fig. 5C).

### AMPK activity in metformin-treated PL-MSCs

**Fig. 6:**
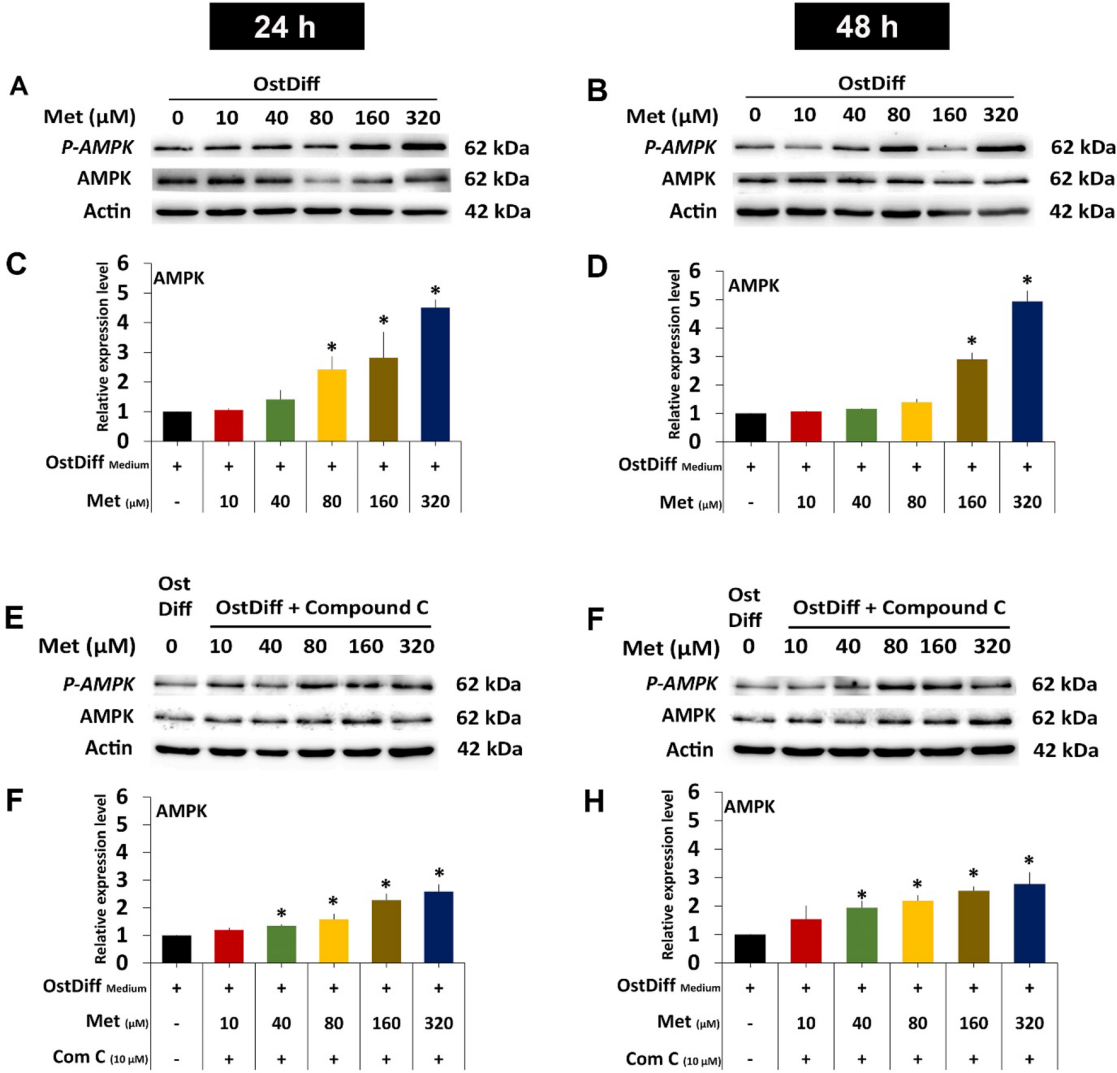
Western blot analysis demonstrated the expression levels of AMPK in PL-MSCs. **A**–**D** The expression levels of AMPK in PL-MSCs treated with metformin at a concentration of 0–320 µM for 24 and 48 h. **E**–**H** The expression levels of AMPK in PL-MSCs treated with 0–320 µM metformin and 10 µM of Compound C for 24 h and 48 h. Each value represents the means ± SEM of three samples. Data are presented as mean ± SEM (n = 3). *p-value ≤ 0.05 compared to PL-MSCs cultured in osteogenic differentiation medium (OstDiff medium) without metformin and Compound C

**Fig. 7:**
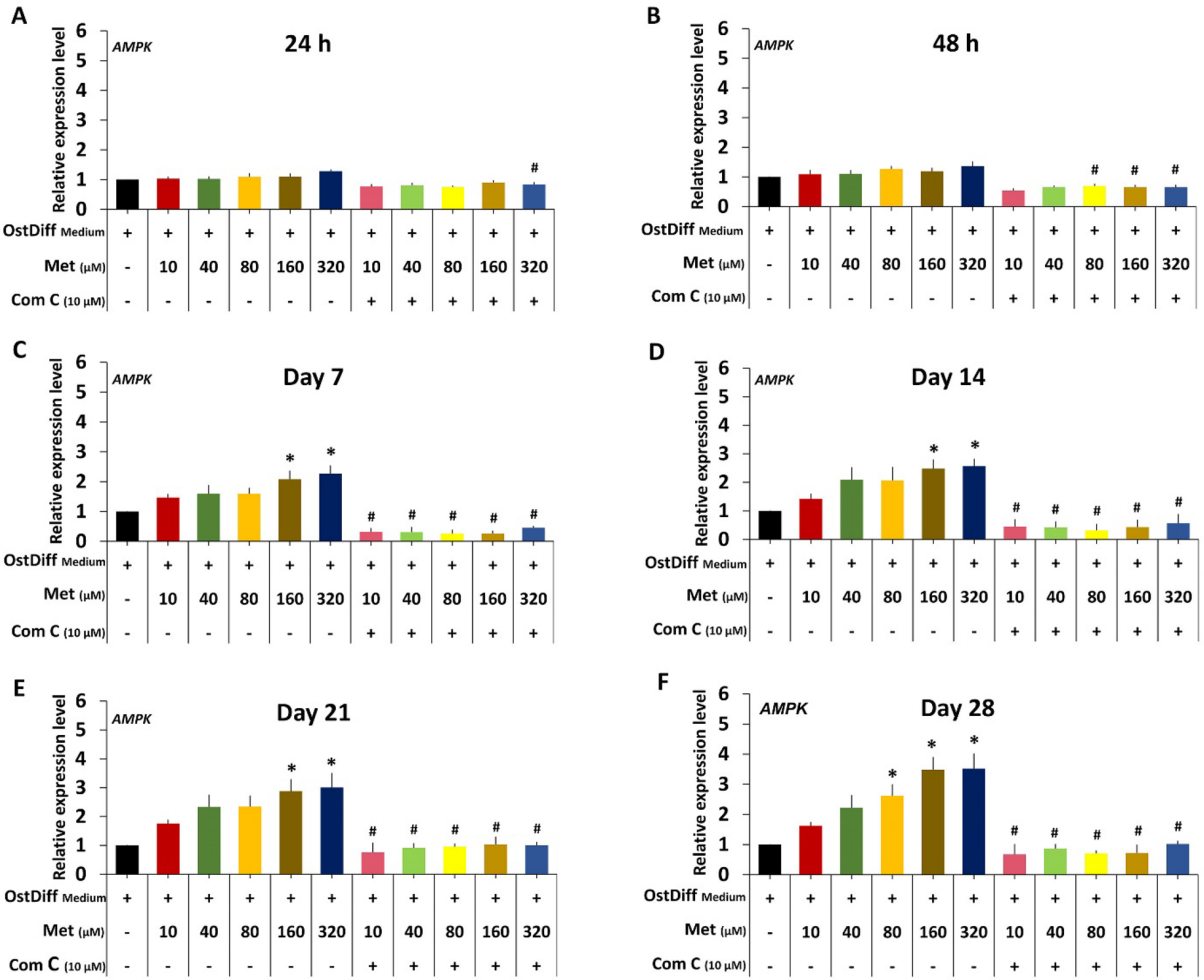
Quantitative real-time RT-PCR demonstrated the expression levels of *AMPK* in PL-MSCs treated with 0–320 µM of metformin and 10 µM of Compound C for 24 h, 48 h, 7 days, 14 days, 21 days and 28 days. Data are presented as mean ± SEM (n = 3). *p-value ≤ 0.05 compared to PL-MSCs cultured in osteogenic differentiation medium (OstDiff medium) without metformin and Compound C. ^#^*p*-value ≤ 0.05 compared to PL-MSCs treated with the same concentration of metformin without Compound C

Based on the data presented above, we next investigate whether metformin enhances osteogenic differentiation of PL-MSCs through the AMPK signaling pathway by determining the levels of AMPK, phosphorylated AMPK, and the pAMPK/AMPK ratio in metformin-treated PL-MSCs using Western blot analysis. The result showed that metformin, at concentrations of 160 and 320 µM, significantly increased the pAMPK/AMPK ratios in PL-MSCs cultured in osteogenic differentiation medium at both 24 (Fig. 6A, C, Supplementary Fig. 1) and 48 h (Fig. 6B, D, Supplementary Fig. 2). Next, we investigated whether Compound C, a specific inhibitor of AMPK, abolished the positive effect of metformin on osteogenic differentiation of PL-MSCs. The results showed that 10 µM Compound C reduced the positive effect of metformin on AMPK activity by significantly reducing the pAMPK/AMPK ratio in PL-MSCs treated with 160 and 320 µM metformin at both 24 and 48 h (Fig. 6E–H, Supplementary Fig. 3, 4). It should be noted that although Compound C decreased AMPK activity in metformin-treated PL-MSCs, it did not completely abolish the positive effect of metformin on AMPK activity (Fig. 6E–H). Consistent with its effect on the pAMPK/AMPK ratio, 10 µM compound C significantly decreased the expression levels of the *AMPK* gene, in PL-MSCs treated with 10–320 µM metformin compared to untreated PL-MSCs cultured with the same concentration of metformin (Fig. 7). These results suggest that metformin increases AMPK activity in PL-MSCs and the addition of compound C, an AMPK inhibitor, reduces the positive effect of metformin on AMPK activity.

### The role of AMPK signaling in osteogenic differentiation of metformin-treated PL-MSCs

**Fig. 8:**
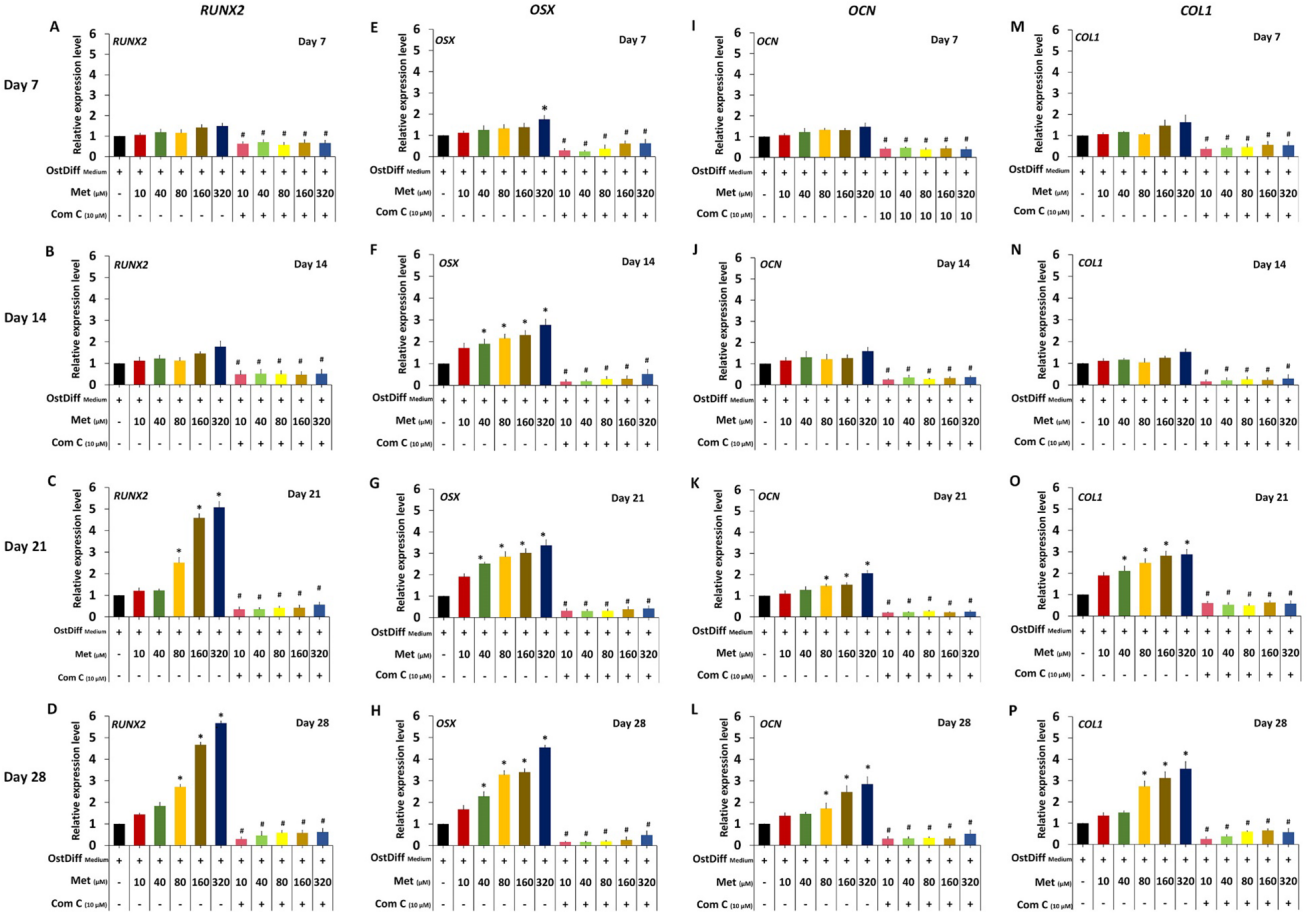
Quantitative real-time RT-PCR demonstrated the expression of osteogenic genes, *RUNX2*, osterix (*OSX*), osteocalcin (*OCN*) and collagen I (*COL1*) in PL-MSCs treated with 0–320 µM of metformin and 10 µM of Compound C on days 7, 14, 21, and 28. Data are presented as mean ± SEM (n = 3). *p-value ≤ 0.05 compared to PL-MSCs cultured in osteogenic differentiation medium (OstDiff medium) without metformin and Compound C. ^#^*p*-value ≤ 0.05 compared to PL-MSCs treated with the same concentration of metformin without Compound C

**Fig. 9:**
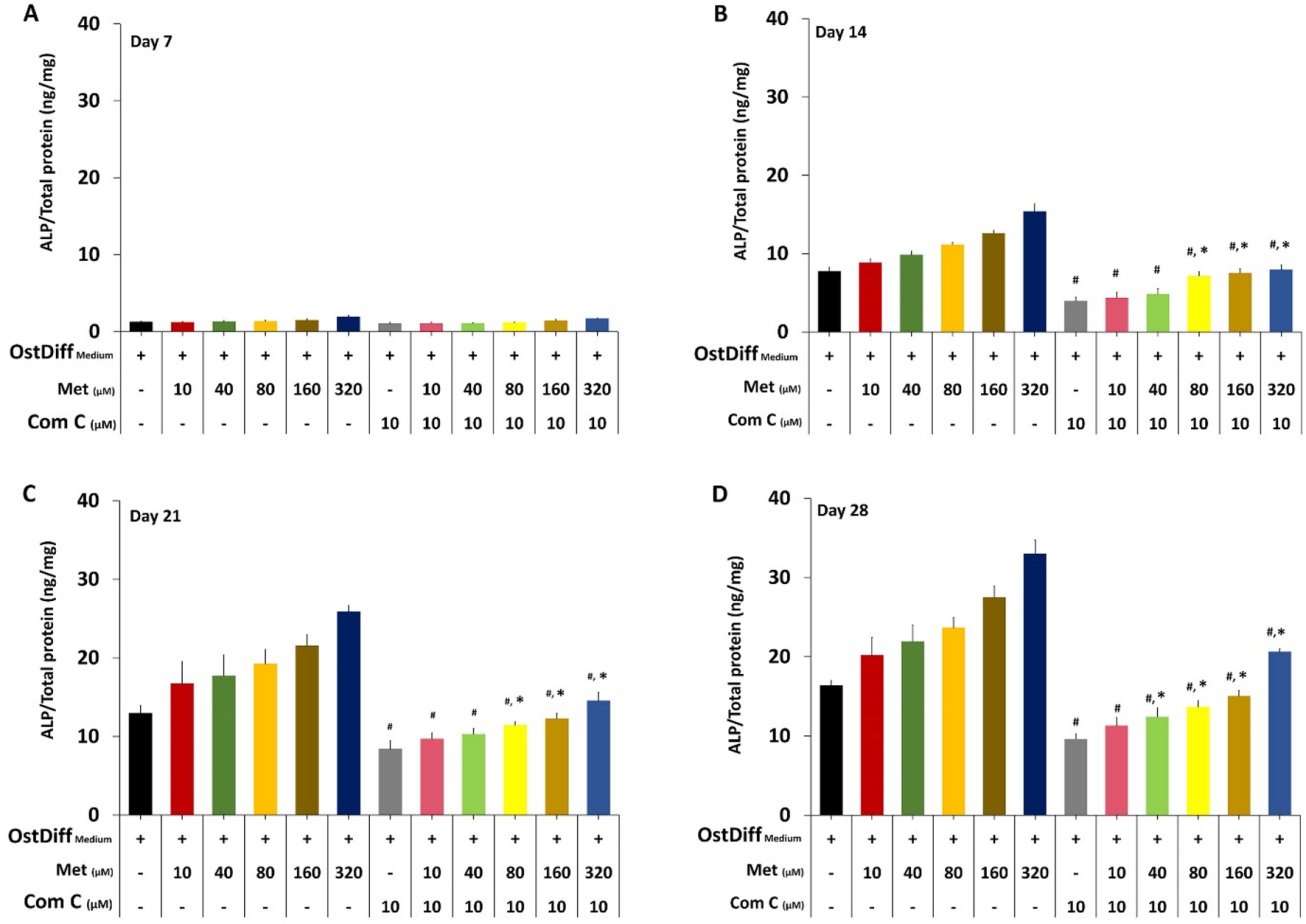
Alkaline phosphatase activity levels of PL-MSCs treated with 0–320 µM of metformin and 10 µM of Compound C on days 7, 14, 21, and 28. Data are presented as mean ± SEM (n = 3). *p-value ≤ 0.05 compared to PL-MSCs cultured in osteogenic differentiation medium (OstDiff medium) without metformin and Compound C. ^#^*p*-value ≤ 0.05 compared to PL-MSCs treated with the same concentration of metformin without Compound C

**Fig. 10:**
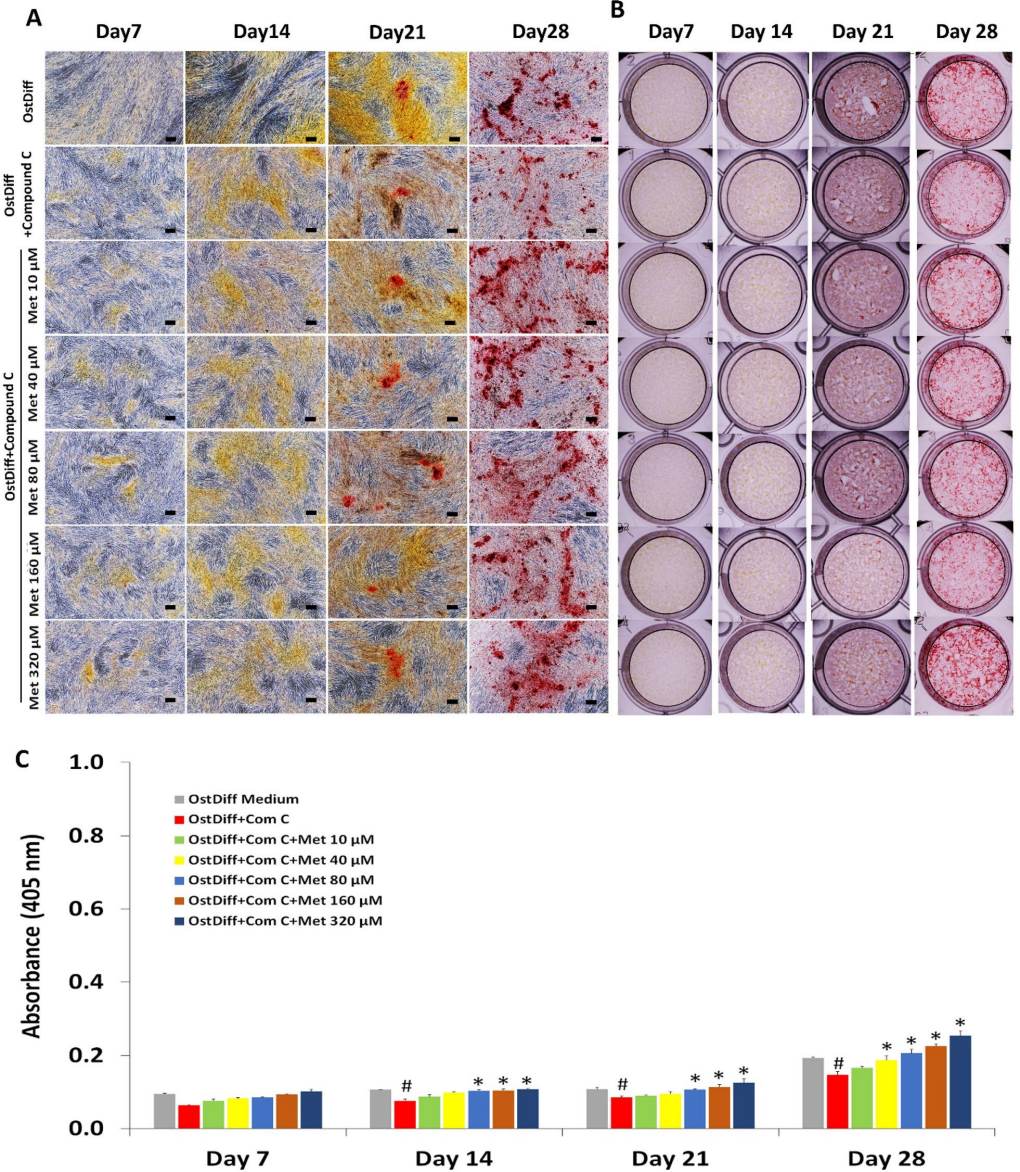
Alizarin Red S staining of metformin-treated PL-MSCs in osteogenic differentiation medium supplemented with Compound C. **A** Positive Alizarin Red S staining of metformin-treated PL-MSCs in osteogenic differentiation medium supplemented with 10 µM of Compound C compared to PL-MSCs cultured in osteogenic differentiation medium supplemented with Compound C without metformin. Scale bar = 100 µm. **B** Whole well imaging of metformin-treated PL-MSCs in osteogenic differentiation medium supplemented with Compound C compared to PL-MSCs cultured in osteogenic differentiation medium supplemented with Compound C without metformin. **C** Quantification of Alizarin Red S staining. Data are presented as mean ± SEM (n = 3). *p-value ≤ 0.05 compared to PL-MSCs cultured in an osteogenic differentiation medium supplemented with Compound C (OstDiff + Com C). ^**#**^*p*-value ≤ 0.05 compared to PL-MSCs cultured in an osteogenic differentiation medium without metformin

To study the role of AMPK signaling in osteogenic differentiation of metformin-treated PL-MSCs, osteogenic differentiation of metformin-treated PL-MSCs in the presence of 10 µM Compound C was evaluated using an osteogenic gene expression assay, an ALP activity assay, and Alizarin Red S staining. The results showed that 10 µM Compound C significantly decreased the expression levels of *RUNX2, OSX, OCN*, and *COL1* genes, in PL-MSCs treated with 10–320 µM metformin compared to PL-MSCs cultured with the same concentrations of metformin without Compound C (Fig. 8). Consistent with the study of gene expression, on culture days 21 and 28, 10 µM Compound C had also significantly reduced ALP activity (Fig. 9) and matrix mineralization (Fig. 10) in PL-MSCs treated with 10–320 µM metformin compared to the untreated PL-MSCs cultured with the same concentration of metformin.

## Discussion

Osteoporosis is one of the most common bone diseases in the elderly [24] that imposes a high cost on the global economy, so an approach to prevent bone loss or accelerate bone repair in these patients is critically needed. Due to their availability, high proliferative capacity, and significant bone-forming capacity, PL-MSCs are considered an attractive cell source for bone regeneration. However, previous studies found inconsistent osteogenic differentiation between isolated PL-MSCs [22, 25] and suggest that an additional strategy is required to improve their bone formation capacity. Metformin, the most widely used hypoglycemic drug in patients with T2DM, has recently gained attention for its role in promoting bone formation [26–28] and reducing the risk of osteoporosis in women without T2DM [29].

Metformin exerts its effects primarily through the activation of AMPK, a critical energy sensor that maintains cellular energy homeostasis. This activation leads to numerous downstream effects that improve energy balance, metabolic health, and bone formation. Metformin inhibits mitochondrial respiratory chain complex I, resulting in decreased ATP production and an increased AMP/ATP ratio within the cell. The increase in the AMP/ATP ratio then activates liver kinase B1 (LKB1), a major upstream kinase that phosphorylates and activates AMPK [30].

Once activated, AMPK initiates a series of downstream effects that enhance catabolic pathways that generate ATP and inhibit anabolic pathways that consume ATP, such as the mechanistic target of rapamycin (mTOR) pathway involved in cell growth and protein synthesis, resulting in the conservation of energy under metabolic stress conditions [31]. Furthermore, AMPK activation increases glucose uptake by increasing the translocation of glucose transporter 4 (GLUT4) to the cell membrane and also increases the phosphorylation and activation of Forkhead box O (FOXO) transcription factors, which regulate several genes involved in oxidative stress response, autophagy, and inhibition of gluconeogenesis [32]. Furthermore, AMPK activation also promotes osteoblast differentiation by upregulating the expression of important transcription factors, such as RUNX2 and Osterix [33], and reduces bone resorption by inhibiting osteoclast formation and activity by suppressing the receptor activator of nuclear factor kappa-Β ligand (RANKL) [34].

Our results show that metformin, at concentrations between 10 and 320 μM, did not show cytotoxic effect on PL-MSCs. This result is consistent with a previous study on MSCs derived from human exfoliated deciduous teeth (SHEDs) showing that 10–400 µM metformin did not affect the viability of SHEDs [35, 36]. Regarding the effect of metformin on osteogenic differentiation, the results show that metformin, at concentrations from 80 to 320 µM, significantly improved the osteogenic differentiation of PL-MSCs, as demonstrated by increased osteogenic gene expression, increased ALP activity, and increased matrix mineralization in a dose- and time-dependent manner. This is consistent with previous studies showing that metformin increased ALP activity and increased bone matrix mineralization in rat BM-MSCs, rat osteosarcoma cell, mouse osteosarcoma cell, and human induced pluripotent stem cells [37–40]. These data suggested that metformin could promote osteogenic differentiation of various stem cells and osteoblastic cells, including PL-MSCs.

The osteogenic differentiation of MSCs is a complicated process that involves various osteogenic genes. RUNX2 and OSX are the first and second critical transcription factors required for osteogenic differentiation, while OCN and COL1 are critical for deposition and mineralization of the bone matrix during the later stages of bone formation [41, 42]. Consistent with its effects on ALP activity and matrix mineralization, 80–320 µM metformin up-regulates the expression levels of *RUNX2, OSX, OCN* and *COL1* genes in PL-MSCs. These results are in agreement with a previous study showing that metformin increased the expression of *RUNX2, OSX,* and *OCN* in SHEDs after osteogenic induction [35].

AMPK, a member of the transferase family, has become one of the most promising potential targets for the prevention and treatment of several diseases due to its various beneficial effects, including anti-aging, antioxidants, anti-inflammatory, and promotion of bone formation [43–46]. Our study shows that the positive effect of metformin on osteogenic differentiation of PL-hMSCs is mediated, at least in part, by increasing AMPK activity, as demonstrated by an increased pAMPK/AMPK ratio in metformin-treated PL-MSCs. Consistent with this, inhibition of AMPK activity by Compound C, a specific AMPK inhibitor, resulted in decreased osteogenic differentiation capacity of metformin-treated PL-MSCs, as evidenced by reduced osteogenic gene expression, ALP activity, and matrix mineralization. The result agrees with a previous study showing that Compound C inhibited metformin-induced AMPK phosphorylation and bone nodule formation in primary osteoblasts derived from rat calvaria [47].

Together, AMPK activation by metformin could promote osteogenic differentiation of PL-MSCs directly by increasing ALP activity, matrix mineralization, and expression levels of osteogenic genes, or indirectly by improving cell energy homeostasis, oxidative stress response, and autophagy by activating FOXO and GLUT4.

## Conclusion

This study demonstrated that metformin, at concentrations of 80–320 µM, increased viability, enhanced osteogenic differentiation, and improved matrix mineralization of PL-MSCs, at least in part, by increasing AMPK activity in these cells. This knowledge highlights the therapeutic potential of metformin beyond glucose control and suggests its use to improve the bone-forming capacity of PL-MSCs for orthopedic bone repair.

## Supplementary Information

Additional file1 (DOCX 1604 kb)

## Data Availability

The data generated or analyzed during this study are included in this article, or, if absent, are available from the corresponding author upon reasonable request.

## Abbreviations

ALP: Alkaline phosphatase
AMPK: Adenosine 5′-monophosphate-activated protein kinase
ANOVA: One-way analysis of variance
BM-MSCs: Bone marrow-derived mesenchymal stem cells
CD: Cluster of differentiation
cDNA: Complementary deoxyribonucleic acid
CO_2_: Carbon dioxide
COL1: Collagen I
DMEM: Dulbecco’s modified Eagle’s medium
DMSO: Dimethyl sulfoxide
DNA: Deoxyribonucleic acid
ECL: Enhanced chemiluminescence
EDTA: Ethylene diamine tetra-acetic acid
FBS: Fetal bovine serum
FITC: Fluorescein isothiocyanate
GAPDH: Glyceraldehyde-3-phosphate dehydrogenase
IOD: Integral optical density
ISCT: International society for cell and gene therapy
MSCs: Mesenchymal stem cells
Ocn: Osteocalcin
Osx: Osterix
p-AMPK: Phosphorylated adenosine 5′-monophosphate-activated protein kinase
PBS: Phosphate buffered saline
PE: Phycoerythrin
PL: Placenta
PL-MSCs: Placenta-derived mesenchymal stem cells
pNPP: P-nitrophenyl phosphate
ROI: Region of interest
RUNX2: Runt-related transcription factor 2
SDS-PAGE: Sodium dodecyl sulfate–polyacrylamide gel electrophoresis
SEM: Standard error of means
qRT-PCR: Quantitative reverse transcription polymerase chain reaction

## References

[1] Hu L, Yin C, Zhao F, Ali A, Ma J, Qian A. Mesenchymal stem cells: cell fate decision to osteoblast or adipocyte and application in osteoporosis treatment. Int J Mol Sci. 2018;19(2):360. 29370110 10.3390/ijms19020360 PMC5855582

[2] Zhang Y, Khan D, Delling J, Tobiasch E. Mechanisms underlying the osteo-and adipo-differentiation of human mesenchymal stem cells. Sci World J. 2012;2012:1–14. 10.1100/2012/793823 PMC3317548 22500143

[3] Al Jofi FE, Ma T, Guo D, Schneider MP, Shu Y, Xu HH, Schneider A. Functional organic cation transporters mediate osteogenic response to metformin in human umbilical cord mesenchymal stromal cells. Cytotherapy. 2018;20(5):650–9. 29555409 10.1016/j.jcyt.2018.02.369 PMC5948160

[4] Bieback K, Brinkmann I. Mesenchymal stromal cells from human perinatal tissues: from biology to cell therapy. World J Stem Cells. 2010;2(4):81. 21607124 10.4252/wjsc.v2.i4.81 PMC3097927

[5] Pinheiro CCG, Bueno DF (2018) Alternative strategies for stem cell osteogenic differentiation. In: Osteogenesis and bone regeneration. IntechOpen

[6] Marupanthorn K, Tantrawatpan C, Kheolamai P, Tantikanlayaporn D, Manochantr S. MicroRNA treatment modulates osteogenic differentiation potential of mesenchymal stem cells derived from human chorion and placenta. Sci Rep. 2021;11(1):7670. 33828198 10.1038/s41598-021-87298-5 PMC8027176

[7] Yousaf Q, Tirmzi A, Ahsan S, Afroz A. Multipotent potential of human adult mesenchymal stem cells. Biochem Mol Biol J. 2018;4(02):1–13.

[8] Kangari P, Talaei-Khozani T, Razeghian-Jahromi I, Razmkhah M. Mesenchymal stem cells: amazing remedies for bone and cartilage defects. Stem Cell Res Ther. 2020;11(1):492. 33225992 10.1186/s13287-020-02001-1 PMC7681994

[9] Wu D, Chang X, Tian J, Kang L, Wu Y, Liu J, Wu X, Huang Y, Gao B, Wang H, et al. Bone mesenchymal stem cells stimulation by magnetic nanoparticles and a static magnetic field: release of exosomal miR-1260a improves osteogenesis and angiogenesis. J Nanobiotechnol. 2021;19(1):209. 10.1186/s12951-021-00958-6 PMC8278669 34256779

[10] Aghayan HR, Salimian F, Abedini A, Fattah Ghazi S, Yunesian M, Alavi-Moghadam S, Makarem J, Majidzadeh AK, Hatamkhani A, Moghri M, et al. Human placenta-derived mesenchymal stem cells transplantation in patients with acute respiratory distress syndrome (ARDS) caused by COVID-19 (phase I clinical trial): safety profile assessment. Stem Cell Res Ther. 2022;13(1):365. 35902979 10.1186/s13287-022-02953-6 PMC9330663

[11] Thaweesapphithak S, Tantrawatpan C, Kheolamai P, Tantikanlayaporn D, Roytrakul S, Manochantr S. Human serum enhances the proliferative capacity and immunomodulatory property of MSCs derived from human placenta and umbilical cord. Stem Cell Res Ther. 2019;10(1):79. 30845980 10.1186/s13287-019-1175-3 PMC6407186

[12] Kangari P, Talaei-Khozani T, Razeghian-Jahromi I, Razmkhah M. Mesenchymal stem cells: amazing remedies for bone and cartilage defects. Stem Cell Res Ther. 2020;11(1):1–21. 33225992 10.1186/s13287-020-02001-1 PMC7681994

[13] Wang L, Ott L, Seshareddy K, Weiss ML, Detamore MS. Musculoskeletal tissue engineering with human umbilical cord mesenchymal stromal cells. Regen Med. 2011;6(1):95–109. 21175290 10.2217/rme.10.98 PMC3057462

[14] Rena G, Hardie DG, Pearson ER. The mechanisms of action of metformin. Diabetologia. 2017;60(9):1577–85. 28776086 10.1007/s00125-017-4342-z PMC5552828

[15] Bailey CJ. Metformin: historical overview. Diabetologia. 2017;60(9):1566–76. 28776081 10.1007/s00125-017-4318-z

[16] Strotmeyer ES, Cauley JA. Diabetes mellitus, bone mineral density, and fracture risk. Curr Opin Endocrinol Diabetes Obes. 2007;14(6):429–35. 17982347 10.1097/MED.0b013e3282f1cba3

[17] Bahrambeigi S, Yousefi B, Rahimi M, Shafiei-Irannejad V. Metformin; an old antidiabetic drug with new potentials in bone disorders. Biomed Pharmacother. 2019;109:1593–601. 30551413 10.1016/j.biopha.2018.11.032

[18] Shah M, Kola B, Bataveljic A, Arnett T, Viollet B, Saxon L, Korbonits M, Chenu C. AMP-activated protein kinase (AMPK) activation regulates in vitro bone formation and bone mass. Bone. 2010;47(2):309–19. 20399918 10.1016/j.bone.2010.04.596 PMC3629687

[19] Kanazawa I, Yamaguchi T, Yano S, Yamauchi M, Sugimoto T. Metformin enhances the differentiation and mineralization of osteoblastic MC3T3-E1 cells via AMP kinase activation as well as eNOS and BMP-2 expression. Biochem Biophys Res Commun. 2008;375(3):414–9. 18721796 10.1016/j.bbrc.2008.08.034

[20] Molinuevo MS, Schurman L, McCarthy AD, Cortizo AM, Tolosa MJ, Gangoiti MV, Arnol V, Sedlinsky C. Effect of metformin on bone marrow progenitor cell differentiation: in vivo and in vitro studies. J Bone Miner Res. 2010;25(2):211–21. 19594306 10.1359/jbmr.090732

[21] Mu W, Liang G, Feng Y, Jiang Y, Qu F. The potential therapeutic role of metformin in diabetic and non-diabetic bone impairment. Pharmaceuticals (Basel). 2022;15(10):1274. 36297386 10.3390/ph15101274 PMC9611301

[22] Manochantr S, Marupanthorn K, Tantrawatpan C, Kheolamai P, Tantikanlayaporn D, Sanguanjit P. The effects of BMP-2, miR-31, miR-106a, and miR-148a on osteogenic differentiation of mscs derived from amnion in comparison with MSCs derived from the bone marrow. Stem Cells Int. 2017;2017:7257628. 29348760 10.1155/2017/7257628 PMC5733904

[23] Dominici M, Le Blanc K, Mueller I, Slaper-Cortenbach I, Marini F, Krause D, Deans R, Keating A, Prockop D, Horwitz E. Minimal criteria for defining multipotent mesenchymal stromal cells. The International Society for Cellular Therapy position statement. Cytotherapy. 2006;8(4):315–7. 16923606 10.1080/14653240600855905

[24] Halvarsson A, Franzen E, Stahle A. Assessing the relative and absolute reliability of the Falls Efficacy Scale-International questionnaire in elderly individuals with increased fall risk and the questionnaire’s convergent validity in elderly women with osteoporosis. Osteoporos Int. 2013;24(6):1853–8. 23124715 10.1007/s00198-012-2197-1

[25] Manochantr S, Up Y, Kheolamai P, Rojphisan S, Chayosumrit M, Tantrawatpan C, Supokawej A, Issaragrisil S. Immunosuppressive properties of mesenchymal stromal cells derived from amnion, placenta, Wharton’s jelly and umbilical cord. Intern Med J. 2013;43(4):430–9. 23176558 10.1111/imj.12044

[26] Sun CK, Weng PW, Chang JZ, Lin YW, Tsuang FY, Lin FH, Tsai TH, Sun JS. Metformin-incorporated gelatin/hydroxyapatite nanofiber scaffold for bone regeneration. Tissue Eng Part A. 2022;28(1–2):1–12. 33971745 10.1089/ten.TEA.2021.0038

[27] Wang P, Ma T, Guo D, Hu K, Shu Y, Xu HHK, Schneider A. Metformin induces osteoblastic differentiation of human induced pluripotent stem cell-derived mesenchymal stem cells. J Tissue Eng Regen Med. 2018;12(2):437–46. 28494141 10.1002/term.2470 PMC5696118

[28] Xie X, Hu L, Mi B, Xue H, Hu Y, Panayi AC, Endo Y, Chen L, Yan C, Lin Z, et al. Metformin alleviates bone loss in ovariectomized mice through inhibition of autophagy of osteoclast precursors mediated by E2F1. Cell Commun Signal. 2022;20(1):165. 36284303 10.1186/s12964-022-00966-5 PMC9594975

[29] Blumel JE, Arteaga E, Aedo S, Arriola-Montenegro J, Lopez M, Martino M, Miranda C, Miranda O, Mostajo D, Nanez M, et al. Metformin use is associated with a lower risk of osteoporosis in adult women independent of type 2 diabetes mellitus and obesity. REDLINC IX study. Gynecol Endocrinol. 2020;36(5):421–5. 31994945 10.1080/09513590.2020.1718092

[30] Hasanvand A. The role of AMPK-dependent pathways in cellular and molecular mechanisms of metformin: a new perspective for treatment and prevention of diseases. Inflammopharmacology. 2022;30(3):775–88. 35419709 10.1007/s10787-022-00980-6 PMC9007580

[31] Green AS, Chapuis N, Maciel TT, Willems L, Lambert M, Arnoult C, Boyer O, Bardet V, Park S, Foretz M, et al. The LKB1/AMPK signaling pathway has tumor suppressor activity in acute myeloid leukemia through the repression of mTOR-dependent oncogenic mRNA translation. Blood. 2010;116(20):4262–73. 20668229 10.1182/blood-2010-02-269837

[32] Yun H, Park S, Kim MJ, Yang WK, Im DU, Yang KR, Hong J, Choe W, Kang I, Kim SS, et al. AMP-activated protein kinase mediates the antioxidant effects of resveratrol through regulation of the transcription factor FoxO1. FEBS J. 2014;281(19):4421–38. 25065674 10.1111/febs.12949

[33] Qin W, Gao X, Ma T, Weir MD, Zou J, Song B, Lin Z, Schneider A, Xu HHK. Metformin enhances the differentiation of dental pulp cells into odontoblasts by activating AMPK signaling. J Endod. 2018;44(4):576–84. 29306537 10.1016/j.joen.2017.11.017 PMC7169970

[34] Shao X, Cao X, Song G, Zhao Y, Shi B. Metformin rescues the MG63 osteoblasts against the effect of high glucose on proliferation. J Diabetes Res. 2014;2014: 453940. 24812633 10.1155/2014/453940 PMC4000639

[35] Zhao X, Pathak JL, Huang W, Zhu C, Li Y, Guan H, Zeng S, Ge L, Shu Y. Metformin enhances osteogenic differentiation of stem cells from human exfoliated deciduous teeth through AMPK pathway. J Tissue Eng Regen Med. 2020;14(12):1869–79. 33049108 10.1002/term.3142

[36] Shen M, Yu H, Jin Y, Mo J, Sui J, Qian X, Chen T. Metformin facilitates osteoblastic differentiation and M2 macrophage polarization by PI3K/AKT/mTOR pathway in human umbilical cord mesenchymal stem cells. Stem Cells Int. 2022;2022:1–12. 10.1155/2022/9498876 PMC9233575 35761829

[37] Gao Y, Xue J, Li X, Jia Y, Hu J. Metformin regulates osteoblast and adipocyte differentiation of rat mesenchymal stem cells. J Pharm Pharmacol. 2008;60(12):1695–700. 19000376 10.1211/jpp.60/12.0017

[38] Cortizo AM, Sedlinsky C, McCarthy AD, Blanco A, Schurman L. Osteogenic actions of the anti-diabetic drug metformin on osteoblasts in culture. Eur J Pharmacol. 2006;536(1–2):38–46. 16564524 10.1016/j.ejphar.2006.02.030

[39] Kanazawa I, Yamaguchi T, Yano S, Yamauchi M, Sugimoto T. Activation of AMP kinase and inhibition of Rho kinase induce the mineralization of osteoblastic MC3T3-E1 cells through endothelial NOS and BMP-2 expression. Am J Physiol Endocrinol Metab. 2009;296(1):E139-146. 19001547 10.1152/ajpendo.90677.2008

[40] Ma J, Zhang Z, Hu X, Wang X, Chen A. Metformin promotes differentiation of human bone marrow derived mesenchymal stem cells into osteoblast via GSK3β inhibition. Eur Rev Med Pharmacol Sci. 2018;22(22):7962–8. 30536344 10.26355/eurrev_201811_16424

[41] Iaquinta MR, Mazzoni E, Bononi I, Rotondo JC, Mazziotta C, Montesi M, Sprio S, Tampieri A, Tognon M, Martini F. Adult stem cells for bone regeneration and repair. Front Cell Dev Biol. 2019;7:268. 31799249 10.3389/fcell.2019.00268 PMC6863062

[42] Sila-Asna M, Bunyaratvej A, Maeda S, Kitaguchi H, Bunyaratavej N. Osteoblast differentiation and bone formation gene expression in strontium-inducing bone marrow mesenchymal stem cell. Kobe J Med Sci. 2007;53(1–2):25–35. 17579299

[43] Garcia D, Shaw RJ. AMPK: mechanisms of cellular energy sensing and restoration of metabolic balance. Mol Cell. 2017;66(6):789–800. 28622524 10.1016/j.molcel.2017.05.032 PMC5553560

[44] Lin SC, Hardie DG. AMPK: sensing glucose as well as cellular energy status. Cell Metab. 2018;27(2):299–313. 29153408 10.1016/j.cmet.2017.10.009

[45] Agius L, Ford BE, Chachra SS. The metformin mechanism on gluconeogenesis and AMPK activation: the metabolite perspective. Int J Mol Sci. 2020;21(9):3240. 32375255 10.3390/ijms21093240 PMC7247334

[46] Zhang R, Liang Q, Kang W, Ge S. Metformin facilitates the proliferation, migration, and osteogenic differentiation of periodontal ligament stem cells in vitro. Cell Biol Int. 2019;44:70. 31293042 10.1002/cbin.11202

[47] Suenaga H, Furukawa KS, Suzuki Y, Takato T, Ushida T. Bone regeneration in calvarial defects in a rat model by implantation of human bone marrow-derived mesenchymal stromal cell spheroids. J Mater Sci Mater Med. 2015;26(11):254. 26449444 10.1007/s10856-015-5591-3 PMC4598349

