# Supplementary material for: Improvement of osteogenic differentiation potential of placenta-derived mesenchymal stem cells by metformin via AMPK pathway activation
Source: Stem Cell Res Ther. 2024 Nov 13;15:417. doi: 10.1186/s13287-024-04014-6 (PMC11559138; doi:10.1186/s13287-024-04014-6)
Supplement: Supplementary file 1 — Additional file1 (DOCX 1604 kb) [file 13287_2024_4014_MOESM1_ESM.docx]

**Supplementary figures and figure legends**

**
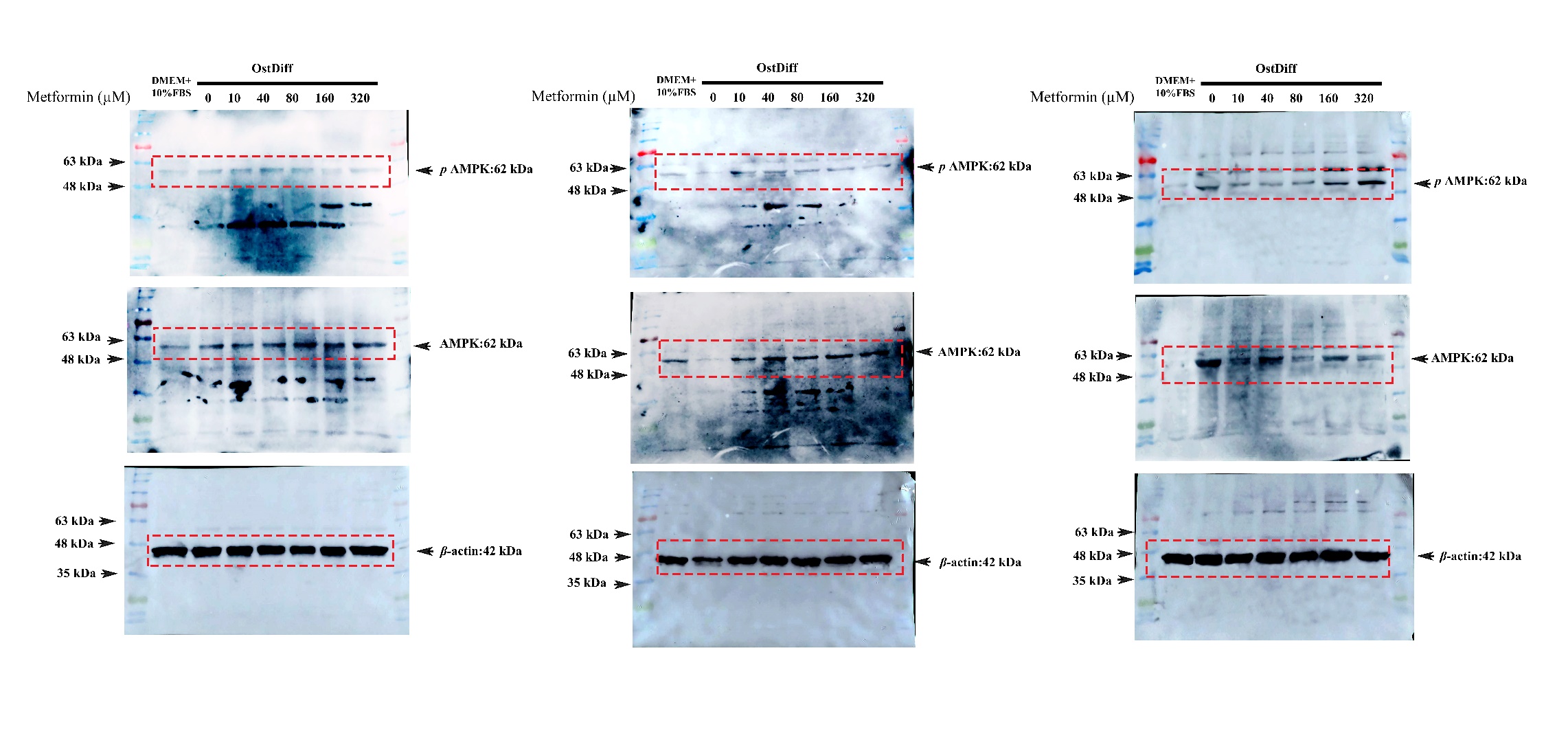
**

**Supplementary Figure 1** Original blots showed the expression of pAMPK/AMPK in PL-MSCs treated with metformin at a concentration of 0-320 µM for 24 h. Three replicate blots were performed. Human β-actin (MW=42 kDa) was used to normalize protein loading.

**
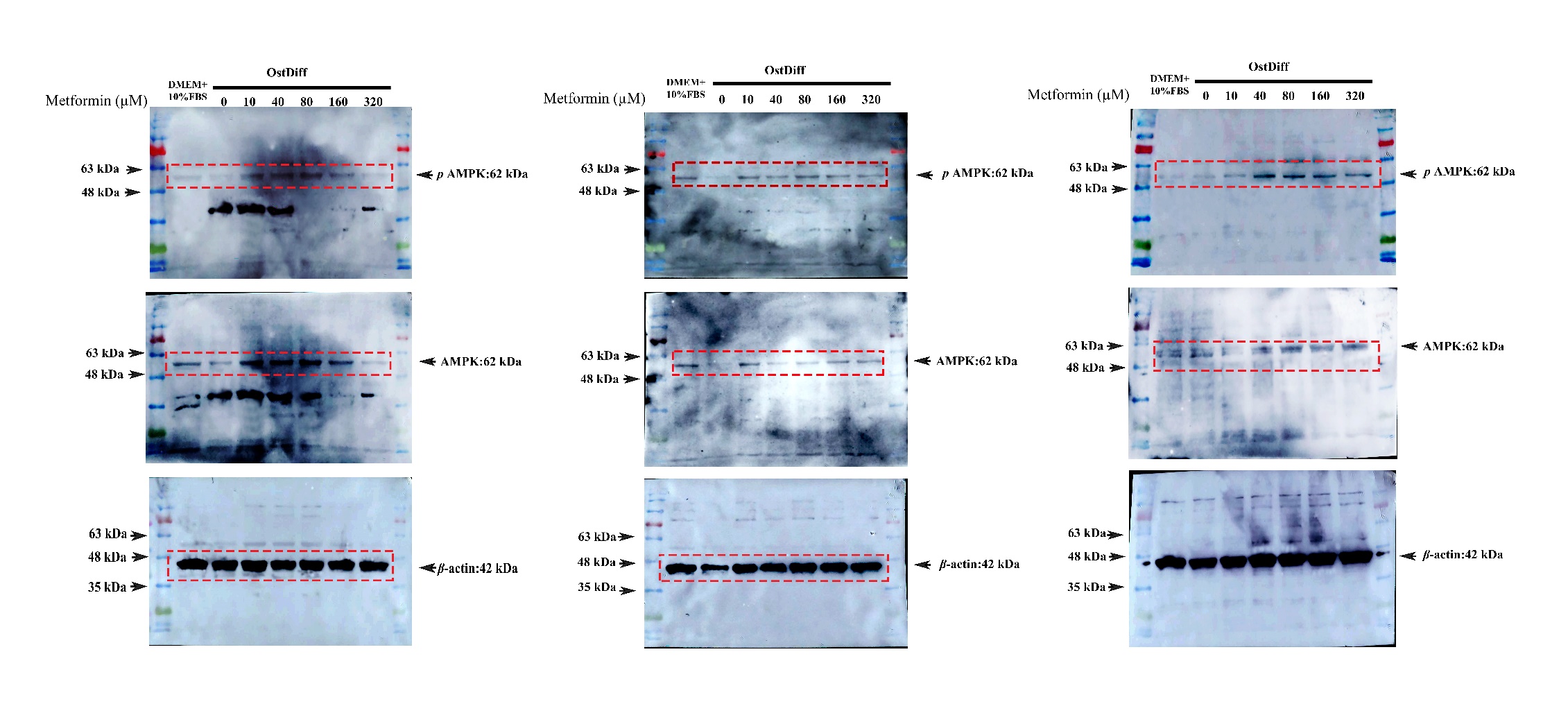
**

**Supplementary Figure 2** Original blots showed the expression of pAMPK/AMPK in PL-MSCs treated with metformin at a concentration of 0-320 µM for 48 h. Three replicate blots were performed. Human β-actin (MW=42 kDa) was used for normalization of protein loading.


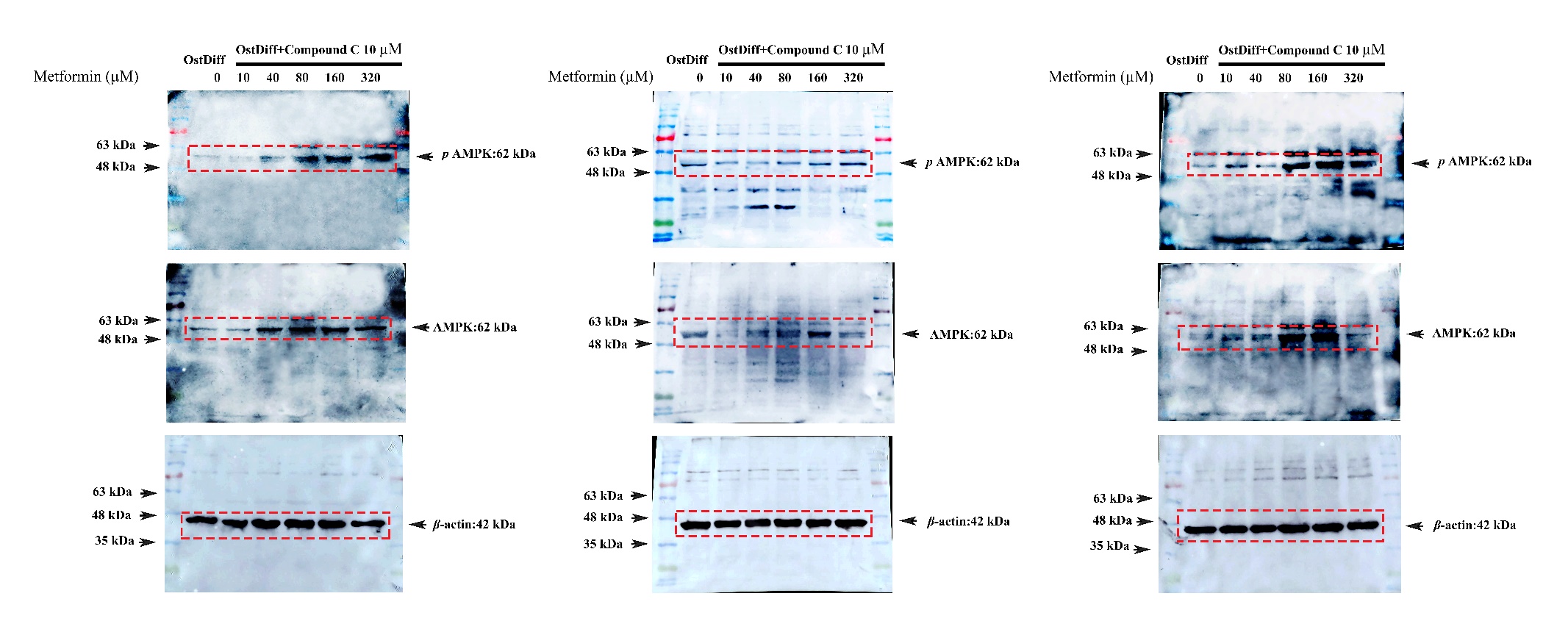


**Supplementary Figure 3:** The Original blots showed the expression of pAMPK/AMPK in PL-MSCs treated with treated with 0-320 µM metformin and 10 µM of Compound C for 24 h. Three replicate blots were performed. Human β-actin (MW=42 kDa) was used to normalize protein loading.

**
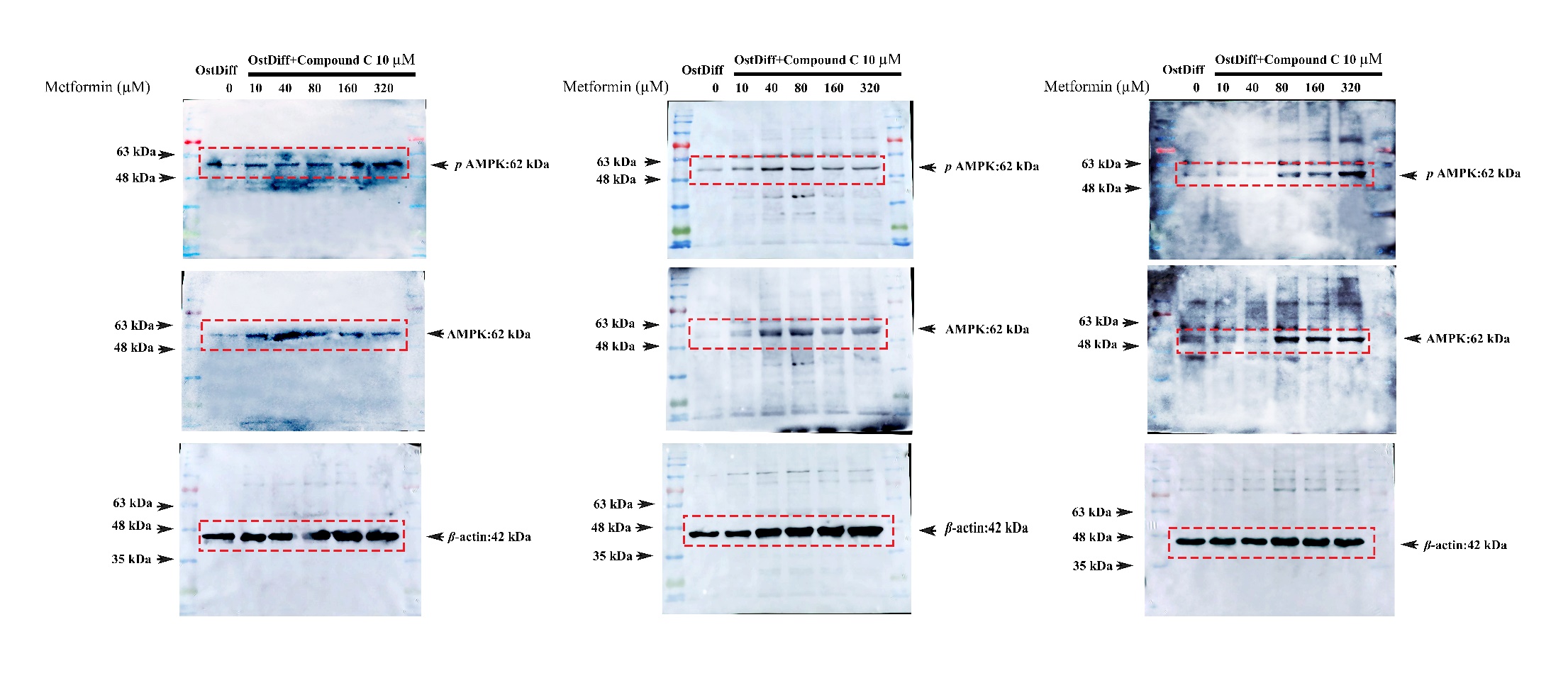
**

**Supplementary Figure 4** Original blots showed the expression of pAMPK/AMPK in PL-MSCs treated with treated with 0-320 µM metformin and 10 µM of Compound C for 48 h. Three replicate blots were performed. Human β-actin (MW=42 kDa) was used to normalize protein loading.
